# Mortise-and-Tenon Connections for Rebar-Free ECC Beams: Experimental and Numerical Investigation

**DOI:** 10.3390/ma19214501

**Published:** 2026-10-26

**Authors:** Manfang Lin, Lingzhi Li, Yunqi Jiang

**Affiliations:** College of Civil Engineering, Tongji University, Shanghai 200092, China

**Keywords:** engineered cementitious composites (ECCs), mortise-and-tenon connection, bending performance, CFRP strengthening, numerical simulation, rebar-free beam

## Abstract

Engineered cementitious composites (ECCs) combine high tensile ductility, multiple cracking, and sustained post-cracking load transfer, making rebar-free structural members feasible. However, eliminating longitudinal reinforcement also removes the conventional load-transfer path across prefabricated components, making reliable connections a key obstacle to their wider application. Mortise-and-tenon joints offer a potential solution through geometric interlocking and local strengthening. This study experimentally investigated one monolithic cast beam and 19 mortise-and-tenon-connected ECC beams considering ECC strength, joint geometry, CFRP strengthening, loading direction, and connection location. A finite-element model was further developed to capture mortise-and-tenon contact, interface degradation, and CFRP strengthening. The results show that mid-span connections strengthened with one and two CFRP layers increased the average peak load by approximately 20% and 47%, respectively, compared to unstrengthened specimens. However, excessive local strengthening may shift damage toward the CFRP termination. Straight joints exhibited smaller loading-direction differences in peak load (1.5–7.4%) than dovetail joints (15.4–21.7%). Moving straight connections from mid-span to the shear span allowed specimens to retain approximately 89–94% of the monolithic reference peak load and 74–90% of its peak deflection. These findings provide a basis for efficient connection design in prefabricated rebar-free ECC structures.

## 1. Introduction

Engineered cementitious composites (ECCs) are characterized by tensile strain-hardening, multiple cracking, and high deformation capacity, which distinguish them from conventional brittle cementitious materials [1]. At the material level, extensive research has been devoted to optimizing fiber systems, matrix composition, and fiber–matrix interactions to improve the mechanical performance and robustness of ECC and related fiber-reinforced cementitious composites [2,3,4]. At the member level, its performance has been investigated in beams, slabs, columns, coupling beams, and shear walls [5,6], while recent studies have further extended ECC to frame and structural-system applications [7,8]. In particular, the sustained tensile resistance provided by fiber bridging enables ECC to maintain load transfer after cracking, creating the possibility of substantially reducing conventional steel reinforcement. Experimental investigations on highly ductile ECC have further demonstrated the feasibility of partially or completely eliminating steel reinforcement in structural members while retaining considerable load-bearing and deformation capacities [9,10]. Nevertheless, existing research has predominantly focused on the behavior of individual ECC members or monolithic structural systems [11]. Comparatively limited attention has been given to the connection of separately fabricated ECC components. This becomes a critical issue for rebar-free construction because eliminating longitudinal reinforcement also removes the conventional steel-based load-transfer path between adjacent components.

The transition from monolithic members to prefabricated or segmented ECC structures therefore requires a connection system capable of restoring structural continuity across component interfaces. Existing research on ECC connections has mainly focused on beam–column joints and precast frame systems, in which ECC is introduced into joint cores, connection zones, or prefabricated members to improve crack control, ductility, shear resistance, and seismic performance [12,13,14]. More recent connection strategies have combined ECC with prefabricated joint configurations or other high-performance materials to further improve damage tolerance and recoverability [15,16]. Although these studies demonstrate the advantages of ECC in connection regions, their structural continuity is still primarily established through longitudinal reinforcement, bar anchorage, mechanical sleeves, or other metallic connectors [15]. Such approaches are therefore not directly applicable to rebar-free ECC components, where no continuous steel reinforcement is available to bridge the discontinuity. A connection mechanism that can transfer forces independently of conventional reinforcement continuity is consequently required.

Mortise-and-tenon connections provide a potential solution by transferring forces through the combined action of adhesive bonding, geometric interlocking, direct bearing, and friction. Inspired by traditional timber construction, mortise-and-tenon concepts have recently been introduced into cement-based prefabricated structures, including concrete shear walls and beam–column joints [17,18]. Related approaches have also been developed for unbonded prestressed concrete joints and geometrically keyed UHPC connections [19,20]. More recently, interlocking tenon systems combined with UHPC have been investigated in prefabricated composite joints [21,22], while the anchorage and pullout behavior of connecting reinforcement within concrete mortise-and-tenon joints has also received attention [23]. Experimental comparisons of dry and epoxy-bonded keyed concrete interfaces indicate that bonding can improve contact uniformity and shear transfer, but its contribution depends on joint geometry and loading conditions [24]. These studies support the potential of combining adhesive bonding with interlocking geometry for prefabricated assembly. Nevertheless, most existing systems still rely on reinforcing bars, prestressing tendons, steel plates, or post-cast materials to maintain overall connection integrity. Moreover, damage concentration around the mortise, tenon, or keyed interface has frequently been observed, indicating that the geometric discontinuity itself may become the governing weak region [18,19,20]. Whether mortise-and-tenon geometry can serve as the primary connection mechanism for rebar-free ECC components while preserving their inherent ductility therefore remains insufficiently understood.

The local weakness introduced by the mortise-and-tenon geometry also creates a need for targeted strengthening. Externally bonded fiber-reinforced polymer (FRP) composites provide an attractive solution because of their high tensile strength, low weight, corrosion resistance, and ease of local application, and have been extensively used to improve the flexural and shear performance of concrete members [25]. Carbon-fiber strengthening has also been successfully applied to ECC–concrete composite members, demonstrating its compatibility with ductile cementitious materials [26,27]. However, strengthening a localized region does not necessarily eliminate the governing weak section. Externally bonded FRP systems can experience intermediate-crack debonding or concrete-cover separation owing to concentrated interfacial stresses [28,29]. In mortise-and-tenon-connected ECC beams, FRP wrapping may therefore suppress opening and damage at the joint while simultaneously creating a local stiffness transition and relocating the critical section toward the boundary of the strengthened region. The effectiveness of FRP should consequently be evaluated not only by the increase in load-bearing capacity, but also by its influence on crack development, deformation capacity, failure localization, and the overall load-transfer mechanism.

Against this background, the present study investigates the flexural behavior of rebar-free cast ECC beams assembled using epoxy-bonded mortise-and-tenon connections. A total of 20 beams, comprising 19 connected specimens and one monolithically cast reference specimen, were tested under four-point bending. The experimental program considered straight and dovetail mortise-and-tenon configurations, zero to two layers of externally bonded CFRP, mid-span and shear-span connection locations, three ECC strength levels, and two loading directions. The effects of these variables on crack evolution, failure mode, load–deflection response, load-bearing capacity, and deformation capacity were systematically examined. A nonlinear finite-element model was subsequently established and validated against the experimental results to further investigate the mechanical response of the connected beams. The distinct contributions are the combined experimental comparison of epoxy-bonded keyed geometries and local CFRP strengthening without continuous rebar, the controlled assessment of joint placement under different bending–shear demands, and an interface-damage model used to interpret the observed shift in critical cracking locations. Rather than considering the mortise-and-tenon joint merely as an assembly detail, this study evaluates the coordinated roles of geometric interlocking, adhesive bonding, and localized FRP strengthening in reconstructing the load-transfer path of rebar-free ECC members. The results provide experimental and numerical evidence for the development of connection strategies that extend the advantages of ECC from monolithic rebar-free members to rapidly assembled structural components.

## 2. Experimental Program

### 2.1. Materials and Mixture Proportions

**Figure 1:**
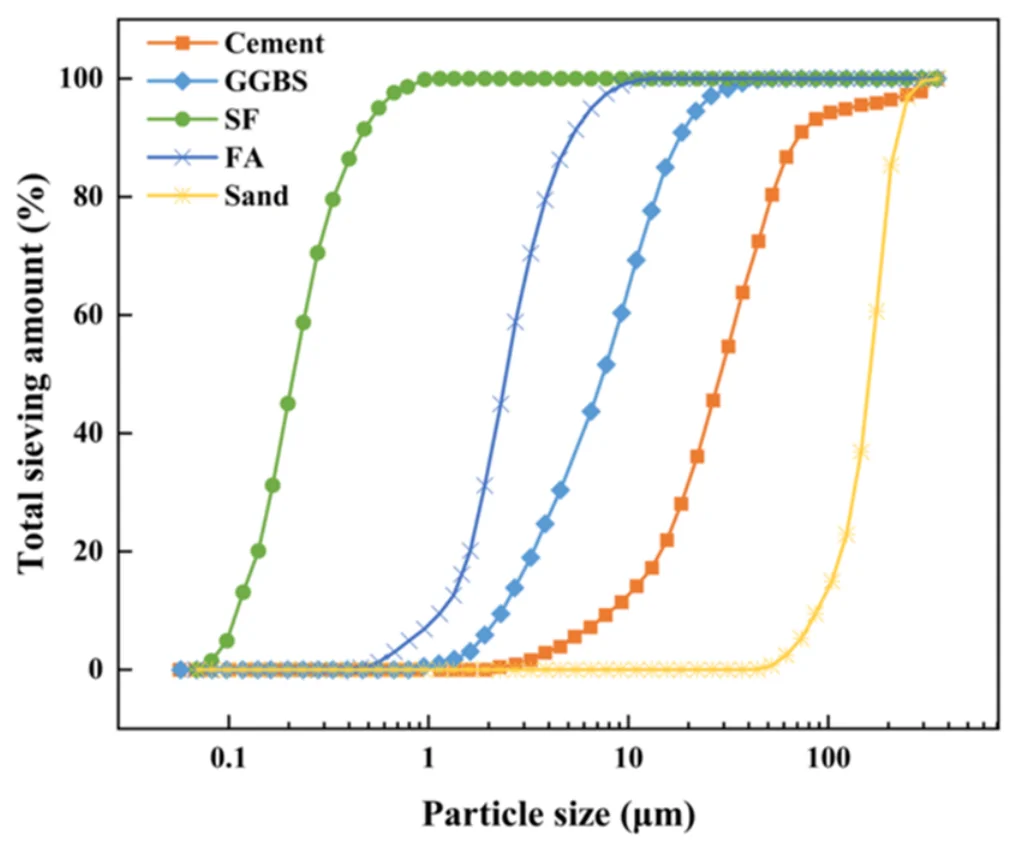
Particle-size distributions of the raw materials.

The mixture proportions of the three ECC mixtures are summarized in Table 1. All mixtures were prepared using P.I 52.5 Portland cement, ground granulated blast-furnace slag (GGBS), fly ash, silica fume, quartz sand, water, a polycarboxylate-based high-range water-reducing admixture, and polyethylene (PE) fibers. Coarse aggregate was excluded to maintain a homogeneous cementitious matrix and facilitate stable fiber dispersion, consistent with common ECC mixture design practice [30]. The particle-size distributions of the principal solid constituents are shown in Figure 1.

Three ECC mixtures, denoted ECC50, ECC75, and ECC100, were designed to provide different strength levels. Their corresponding water-to-binder ratios (w/b) were 0.40, 0.27, and 0.16, respectively. The variations in cementitious-material content and w/b were used to obtain the required range of compressive and tensile strengths while maintaining strain-hardening behavior. This range permits an assessment of the strength–ductility compromise relevant to different structural demands: lower-strength ECC can offer greater deformation capacity, whereas higher-strength ECC may be advantageous where joint bearing and member resistance are critical. The PE fibers had a nominal diameter of 0.024 mm, a length of 18 mm, a density of 900 kg/m^3^, and a nominal tensile strength of 1500 MPa.

**Figure 2:**
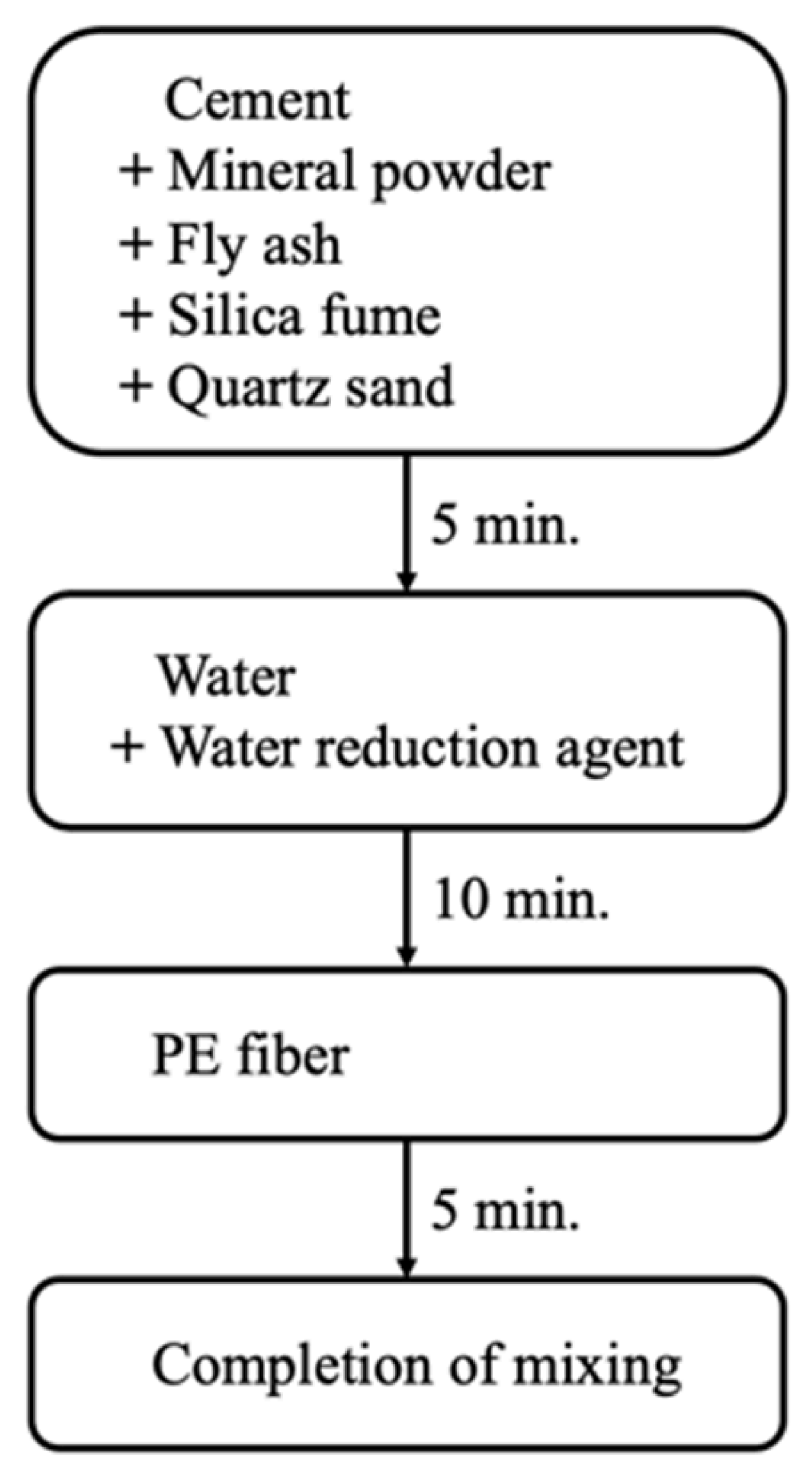
Mixing procedure of ECC.

Because of the relatively low w/b and high powder content of the ECC mixtures, a controlled mixing procedure was adopted, as illustrated in Figure 2. The cement, GGBS, fly ash, silica fume, and quartz sand were first dry-mixed for 5 min. Water and the water-reducing admixture were subsequently added and mixed for another 10 min until a homogeneous and sufficiently fluid matrix was obtained. Finally, the PE fibers were gradually introduced and mixed for an additional 5 min to promote uniform fiber dispersion. All specimens were maintained at room temperature and tested at approximately 28 days after casting.

### 2.2. Mechanical Properties of ECC

**Figure 3:**
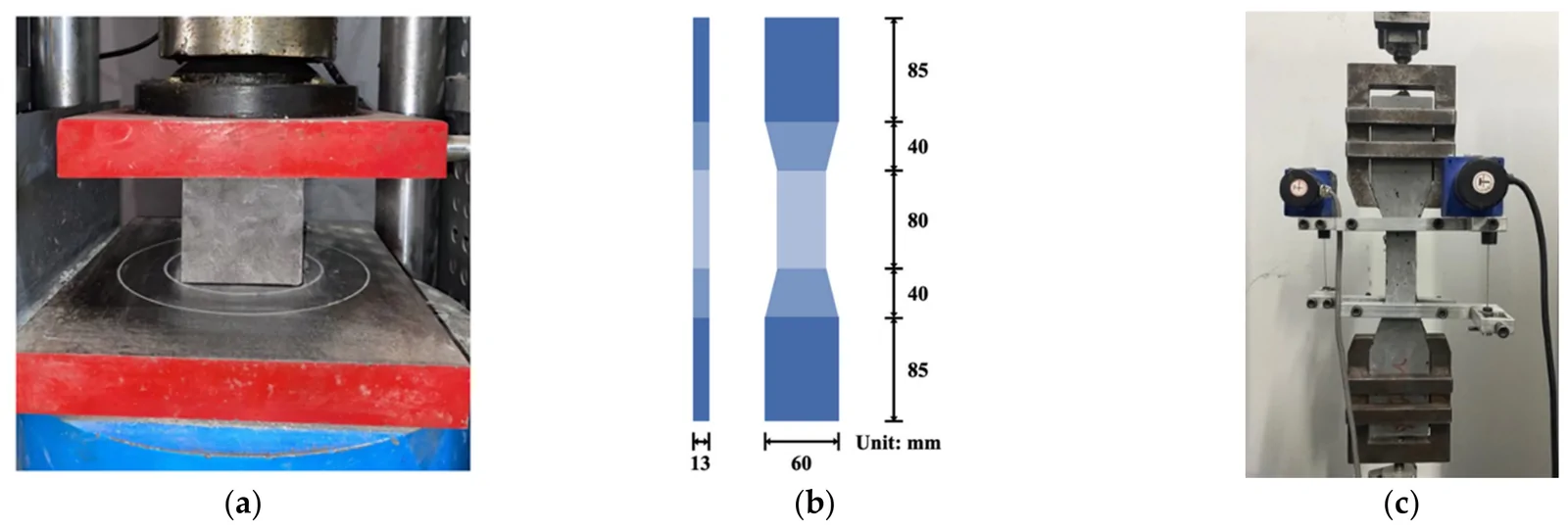
Test device for ECC properties: (**a**) compressive test setup; (**b**) dog-bone specimen; (**c**) direct tensile test setup.

**Figure 4:**
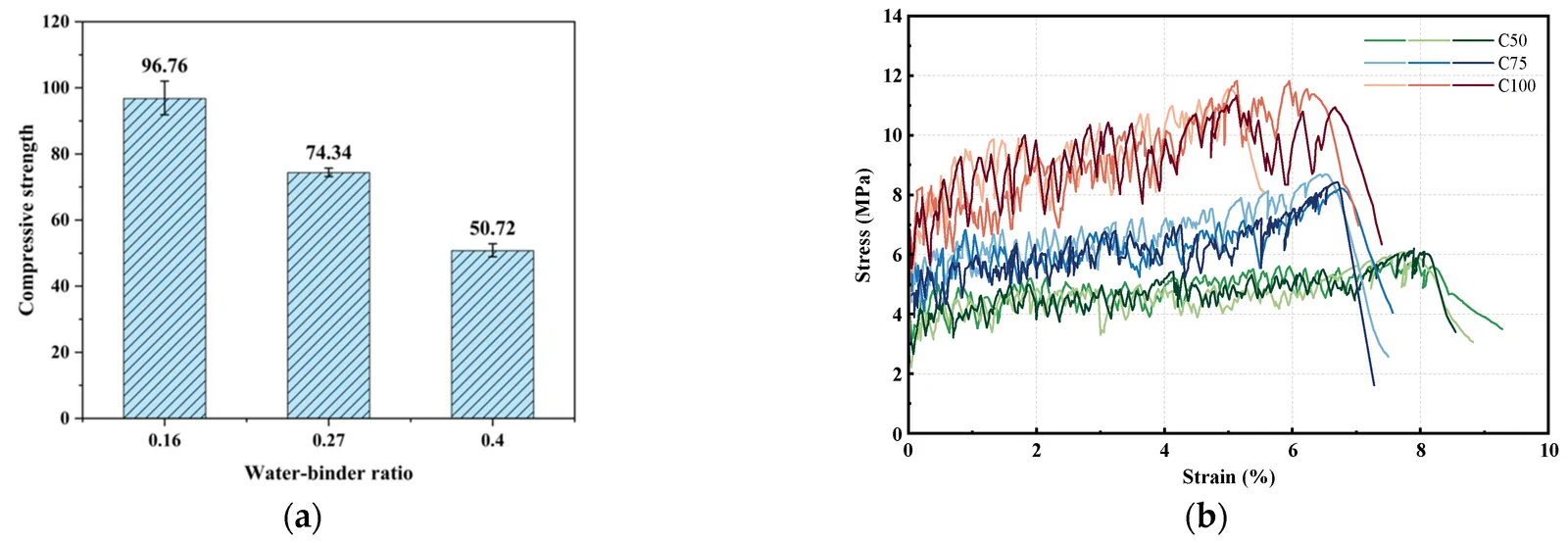
Mechanical properties of ECC: (**a**) compressive strength; (**b**) tensile stress–strain curves.

The compressive and direct tensile properties of the three ECC mixtures were determined in accordance with JC/T 2461-2018 [31]. Three specimens were tested for each mixture and each mechanical property, and their means and sample standard deviations are summarized in Table 2. The compressive strength was measured using 100 × 100 × 100 mm^3^ cubic specimens, as shown in Figure 3a. Before testing, the loading surfaces were cleaned to ensure uniform contact with the platens. The specimens were centered in a 3000 kN compression testing machine and loaded at a rate of 1.0 MPa/s until failure. The measured mean compressive strengths were 50.72, 74.34, and 96.76 MPa for ECC50, ECC75, and ECC100, respectively. As shown in Figure 4a, the compressive strength increased progressively as the w/b decreased from 0.40 to 0.16.

Direct tensile tests were conducted using dog-bone specimens with a gauge region 80 mm long, 30 mm wide, and 13 mm thick, as illustrated in Figure 3b. Three specimens were tested for each ECC mixture under displacement control at a loading rate of 1 mm/min. Two displacement gauges were installed on opposite sides of the gauge region to record axial deformation, while the tensile force was measured by the load cell of the testing machine, as shown in Figure 3c.

The tensile stress–strain responses of all specimens are presented in Figure 4b. Increasing the ECC strength level resulted in higher tensile resistance but a progressively reduced tensile deformation capacity. This strength–deformation trade-off is subsequently reflected in the flexural responses of the connected beams. For rebar-free connected beams, ECC grade selection should therefore consider both the required load resistance and the tensile deformation capacity needed to accommodate distributed cracking near the connection.

### 2.3. Beam Specimens and Test Setup

Twenty rebar-free ECC beams were tested, including 19 mortise-and-tenon-connected specimens and one monolithically cast reference beam. The tested variables were as follows: joint connection type (straight, dovetail), number of CFRP layers (0, 1, and 2 layers), connection location (shear span, mid-span), ECC strength (low, medium, and high strength), and loading direction (X, Y). Table 3 lists the specimens. One beam was tested for each structural configuration; the matrix was organized into controlled comparison subsets, not a fully replicated factorial design. All beams were fabricated and tested using consistent nominal dimensions and procedures, which supports direct within-program comparisons but does not establish specimen-to-specimen scatter or statistical significance.

**Figure 5:**
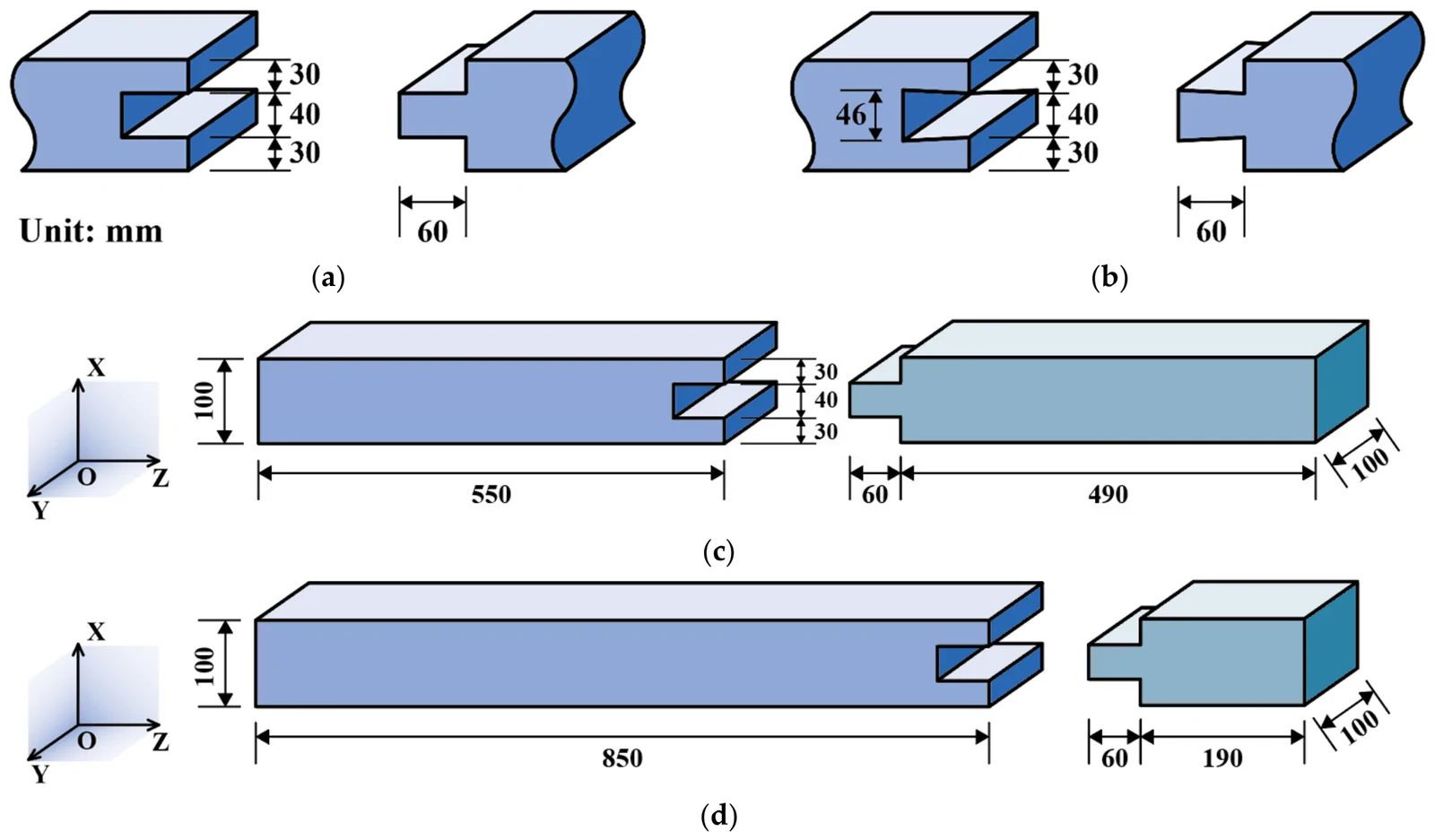
Specimen dimensions: (**a**) straight mortise-and-tenon joint; (**b**) dovetail mortise-and-tenon joint; (**c**) connection located at mid-span; (**d**) connection located at shear span.

All beams had an overall length of 1040 mm and a rectangular cross-section of 100 × 100 mm^2^. The clear span was 900 mm, and the two loading points were separated by 300 mm. Straight and dovetail joint geometries were selected to isolate the effect of inclined versus prismatic contact surfaces while retaining comparable overall connection depth and a nominal 60 mm tenon length (Figure 5). The straight joint used 30 mm upper and lower ligaments and a 40 mm central tongue, whereas the dovetail configuration introduced 46 mm of local keyed depth, with 30 mm edge ligaments. These dimensions were selected as laboratory-scale geometries compatible with the 100 mm section and assembly requirements. The dimensions shown in Figure 5 are nominal design values, and the actual dimensional deviations of the cast joints were not quantified.

**Figure 6:**
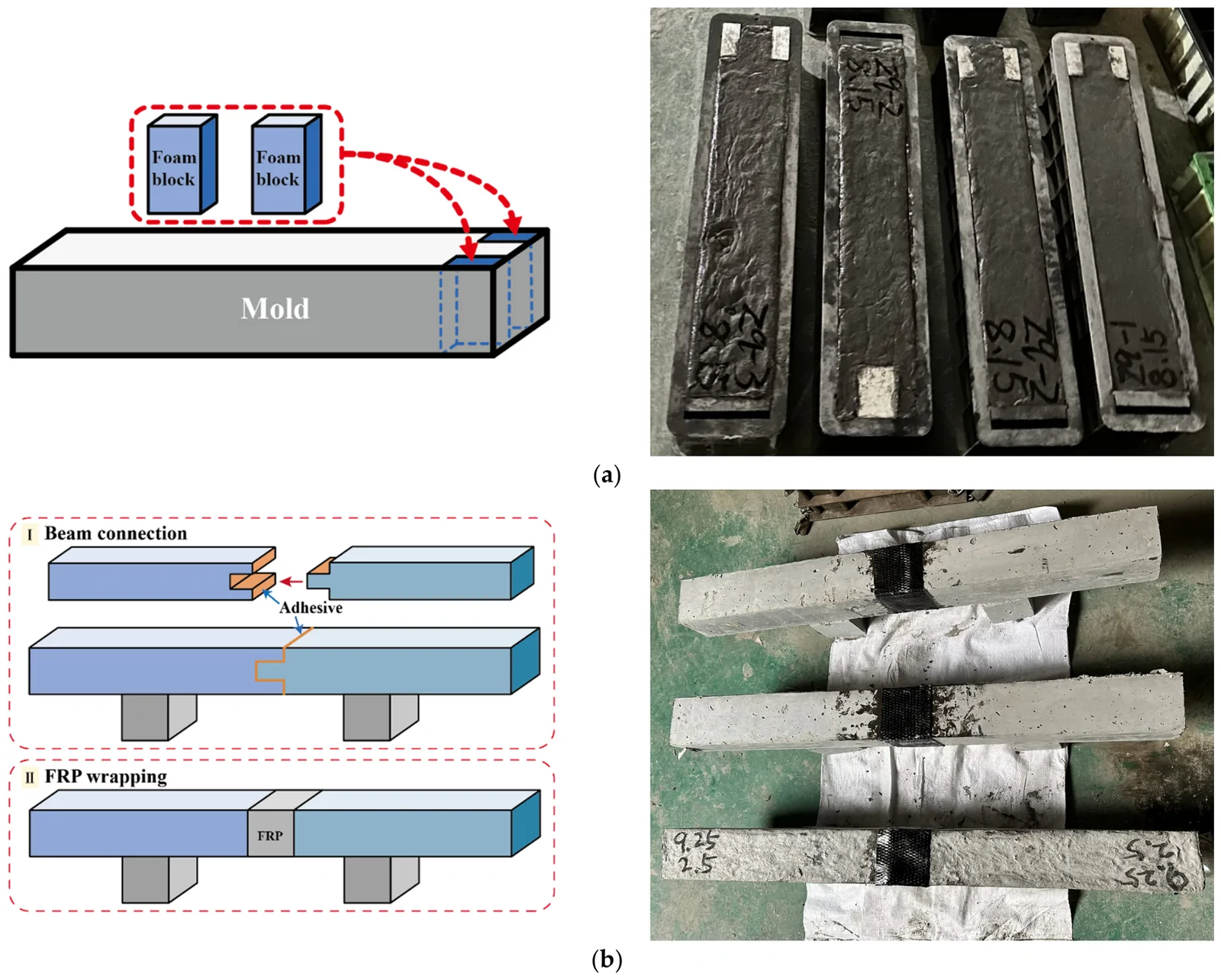
Fabrication and processing of mortise-and-tenon-connected beams: (**a**) fabrication of cast ECC segments; (**b**) connection assembly and CFRP wrapping.

The connected specimens were fabricated as two separate ECC segments. Approximately seven days after casting, the ECC beam segments were demolded and assembled. The mating surfaces of the mortise-and-tenon joints were polished with sandpaper and cleaned with acetone before bonding with Sikadur-330 CN, a two-component epoxy adhesive. Components A and B were mixed at an A:B weight ratio of 3:1, in accordance with the manufacturer’s product data sheet for Sikadur-330 CN. Uniform pressure was applied during assembly to maintain an adhesive layer thickness of approximately 0.5–1.0 mm. The reported product properties include a tensile strength of at least 40 MPa, a tensile modulus of at least 3.0 GPa, and a tensile bond strength to concrete of at least 2.5 MPa. According to the supplier-provided specifications, the adhesive has a working time of approximately 60 min at 25 °C and a curing time of 24 h. The assembled specimens were maintained at room temperature and tested approximately one month after casting, allowing approximately three weeks for adhesive curing. Externally bonded FRP strengthening has been widely applied to improve the flexural performance and damage resistance of structural members [32,33]. Accordingly, local CFRP wrapping was applied to selected specimens in this study to restrain joint opening and enhance force transfer across the mortise-and-tenon connection. The CFRP sheet had a width of 100 mm and a nominal thickness of 0.167 mm per layer. The carbon-fiber fabric had an elastic modulus of approximately 230 GPa, a tensile strength of 3400 MPa, and an ultimate tensile strain of 1.5%, and exhibited brittle tensile failure without a yield plateau. In accordance with GB 50728-2011 [34], the material was classified as Class I carbon-fiber cloth. Depending on the specimen group, zero, one, or two CFRP layers were applied using Sikadur-330 CN epoxy adhesive. The unidirectional carbon fibers were aligned parallel to the longitudinal axis of the beam to resist flexural tension. Each CFRP sheet was centered over the mortise-and-tenon connection, covering a length of 100 mm along the beam axis. The sheets were wrapped around the beam cross-section for one or two complete turns, corresponding to the designated number of CFRP layers. Wrapping started at an edge of the upper surface and ended with a 100 × 100 mm^2^ overlap on the same surface to provide anchorage. After strengthening, the specimens were maintained at room temperature until testing at approximately 28 days after casting. The fabrication and strengthening procedures are illustrated in Figure 6.

The experimental matrix was designed primarily around the ECC75 specimens, while ECC50 and ECC100 beams were additionally tested to examine the influence of material strength. Most connections were placed at mid-span, where the bending moment was maximum, to investigate the behavior of the joint under the most critical flexural demand. Additional straight-joint specimens with connections located in the shear span were tested to evaluate whether relocating the geometric discontinuity away from the maximum-moment region could improve the global flexural response. The mid-span connections were located 450 mm from each support, whereas the shear-span connections were located 150 and 750 mm from the two supports, respectively. Both X- and Y-direction loading configurations were considered because the mortise-and-tenon geometry is not identical in the two transverse directions. As defined in Figure 5c, X- and Y-direction loadings correspond to applying the transverse load along the two orthogonal axes of the beam cross-section by rotating the joint orientation by 90° about the longitudinal axis.

A unified designation system was adopted to identify the experimental variables. For example, C75-SX1-M denotes an ECC75 beam with a straight mortise-and-tenon joint (S), X-direction loading (X), one CFRP layer (1), and a mid-span connection (M). Correspondingly, D denotes a dovetail joint, Y represents Y-direction loading, and S in the final position denotes a shear-span connection. The monolithically cast ECC75 reference beam is designated C75-REF.

**Figure 7:**
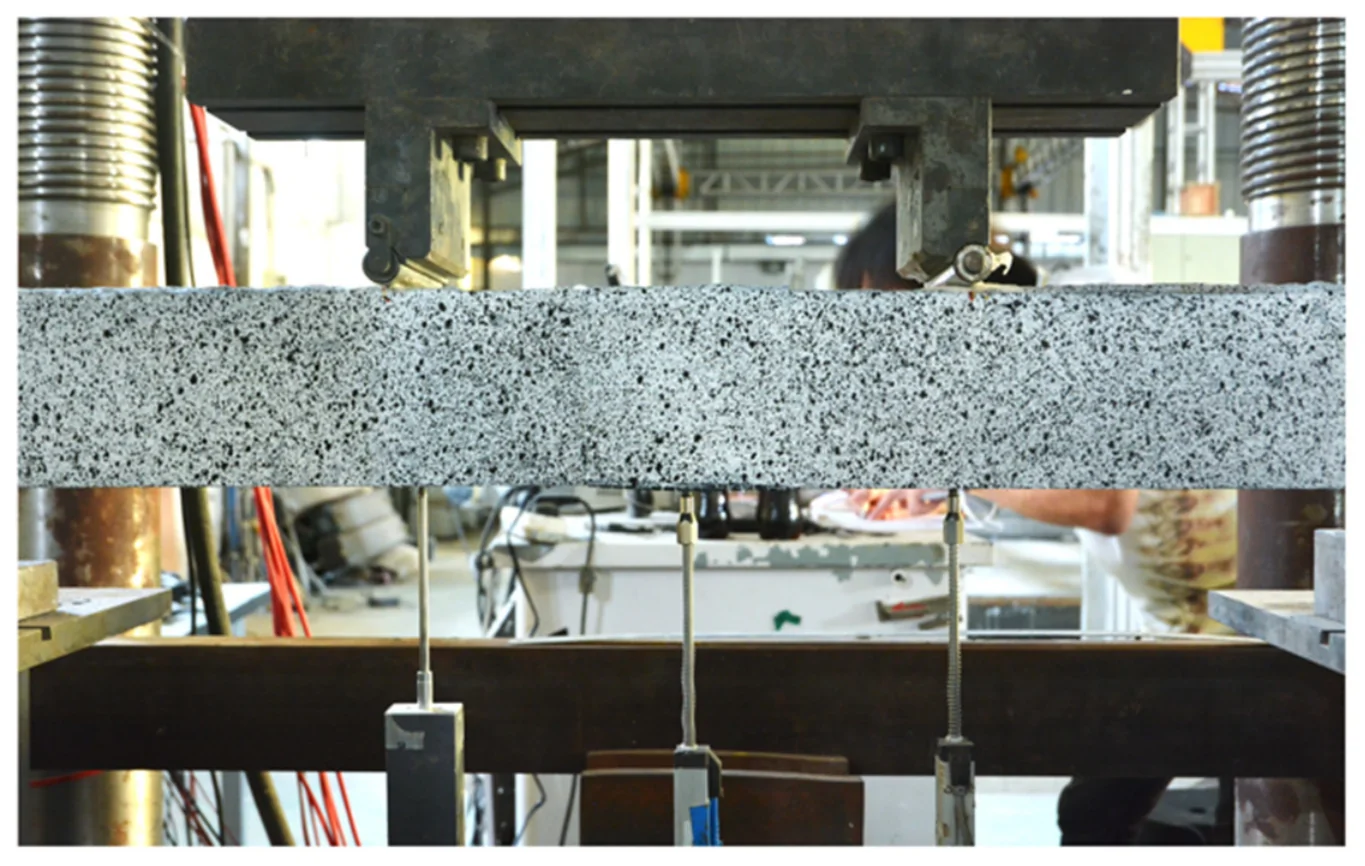
Four-point bending test setup.

Four-point bending tests were conducted using an electro-hydraulic servo testing system with a maximum loading capacity of 3000 kN, as shown in Figure 7. Displacement-controlled loading was applied at a rate of 1 mm/min. Load and deflection were recorded continuously during testing. Displacement gauges were installed at the loading and mid-span positions to determine the vertical deformation of the specimens. In addition, the beam surface was photographed at 10 s intervals throughout loading, and the recorded images were subsequently processed using digital image correlation (DIC) to assist in identifying the evolution and localization of cracking. The specimen surfaces were prepared with manually applied black speckles on a white matte background; the camera resolution was 5184 × 3456 pixels, and image correlation used a nominal subset of 31 × 31 pixels and a 5-pixel step. The DIC maps are used to visualize relative localization and are not treated as independently validated absolute strain measurements.

## 3. Test Results and Discussion

### 3.1. Overall Flexural Response and Failure Evolution

**Figure 8:**
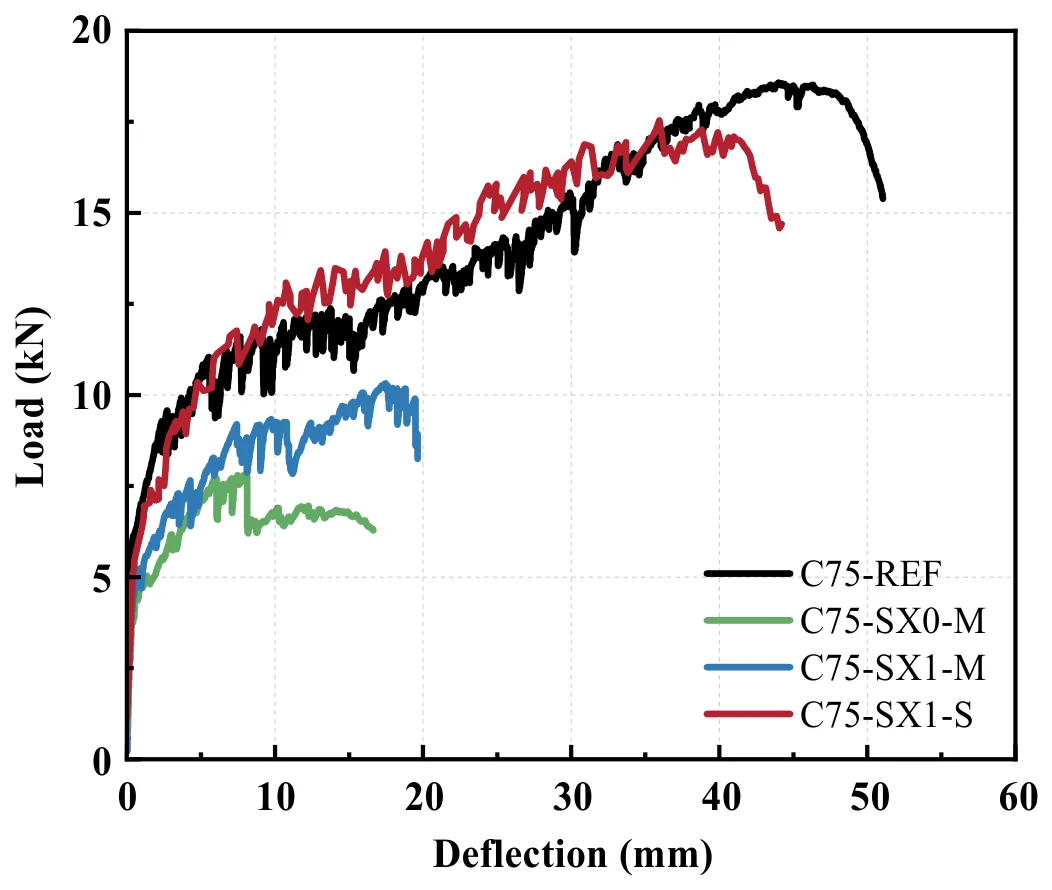
Load–deflection curves of representative specimens.

**Figure 9:**
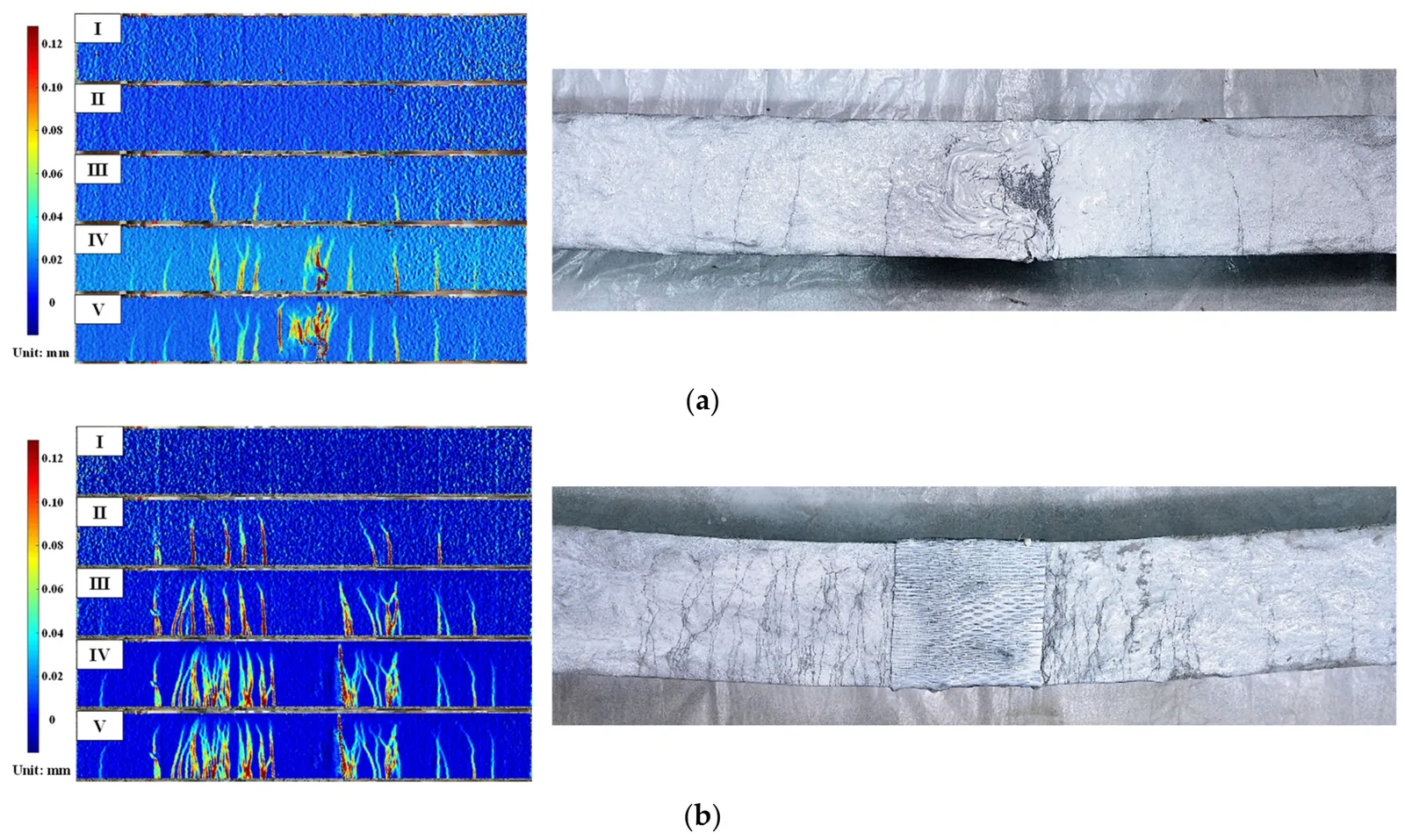

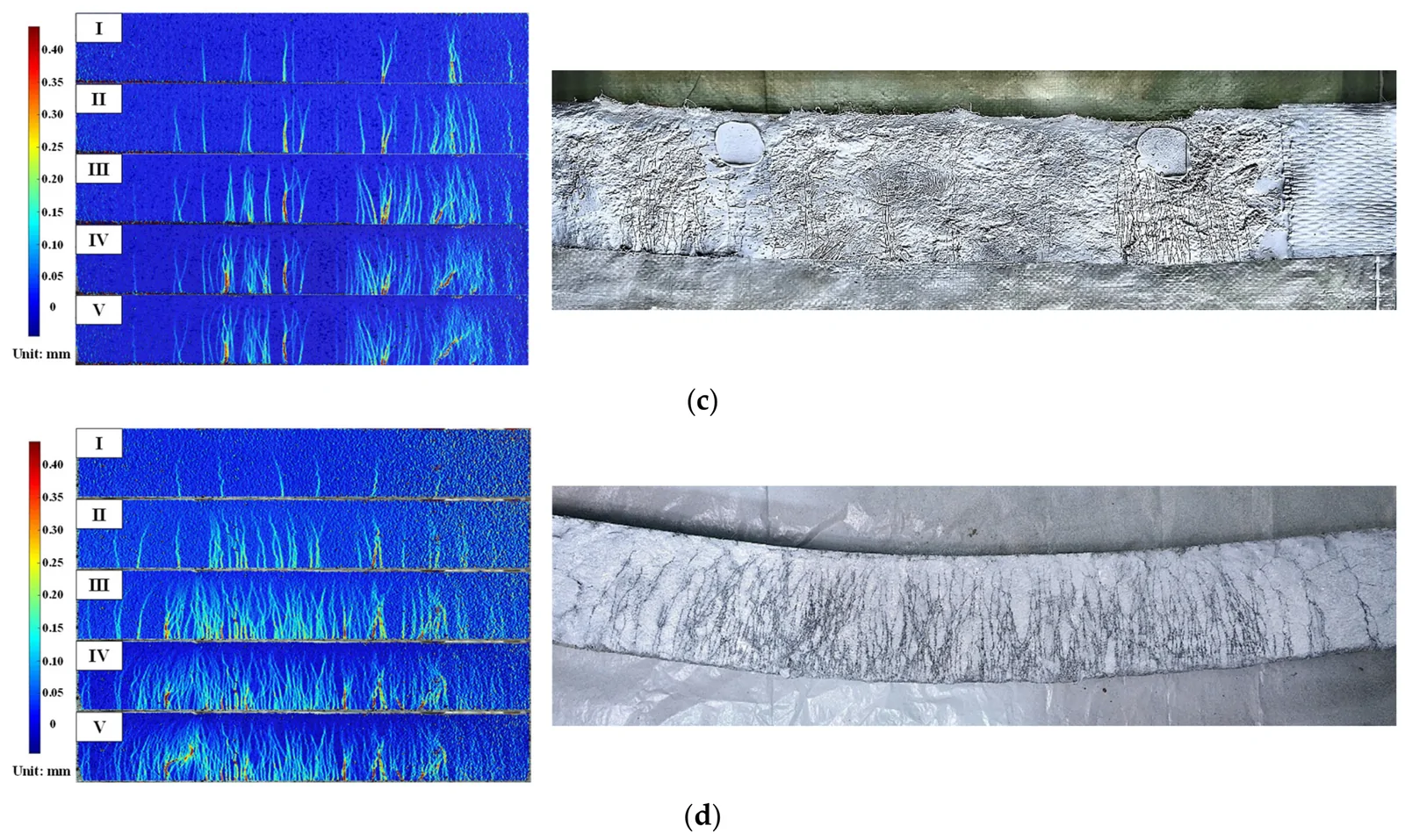
Crack patterns of beams: (**a**) C75-SX0-M; (**b**) C75-SX1-M; (**c**) C75-SX1-S; (**d**) C75-REF.

Figure 8 and Figure 9 jointly illustrate the crack evolution, failure characteristics, and load–deflection responses of representative beams. To correlate the structural response with the development of cracking, the loading process was divided into five characteristic stages. Stages I–IV correspond to 40%, 60%, 80%, and 100% of the peak load on the ascending branch, respectively, while Stage V represents the final recorded post-peak state. The corresponding loads and mid-span deflections of the four representative specimens are summarized in Table 4. The color scales in Figure 9 indicate the DIC-estimated local crack-opening magnitudes (mm), allowing crack development to be visualized at different loading stages. Together with the corresponding mid-span deflections in Table 4, these results characterize both local cracking and global deformation during the loading process.

At the initial loading stage, all specimens exhibited an approximately linear load–deflection response and no significant deterioration in stiffness was observed. For the mid-span-connected specimens, cracking was initially restrained around the mortise-and-tenon region because the bonded connection maintained local continuity before appreciable interface damage occurred. By comparison, fine flexural cracks appeared earlier in the continuous bending region of the shear-span-connected and monolithic beams.

As the load increased, the specimens entered a pronounced post-cracking hardening stage. The load continued to increase despite progressive stiffness degradation, reflecting the multiple-cracking and fiber-bridging characteristics of ECC. However, the spatial development of cracking differed markedly among the four representative configurations.

For the unstrengthened mid-span-connected beam, cracking gradually concentrated around the mortise-and-tenon region. Once the adhesive interface began to deteriorate, additional deformation was increasingly accommodated by the joint rather than being distributed along the beam. Consequently, the load–deflection curve exhibited a relatively short hardening stage, and the connection progressively developed into the governing weak region.

The addition of CFRP substantially altered this response. In the CFRP-strengthened mid-span beam, opening of the original mortise-and-tenon connection was effectively restrained, and multiple cracks developed in the adjacent ECC instead of localizing directly within the joint. This allowed the specimen to sustain a longer post-cracking load-development stage. With increasing deformation, the cracks gradually concentrated in the ECC immediately adjacent to the CFRP termination. The dominant failure region therefore shifted from the original connection to the boundary between the strengthened and unstrengthened beam segments. The CFRP remained bonded to the ECC, with no obvious macroscopic debonding or peeling observed.

A further change occurred when the strengthened connection was moved from the mid-span region to the shear span. The pure bending region then remained continuous, allowing multiple flexural cracks to develop over a much larger portion of the beam. Correspondingly, the load–deflection response exhibited a substantially extended hardening stage and approached that of the monolithically cast reference specimen. The connection itself no longer governed the global response because it was located away from the region of maximum bending moment.

The monolithic reference beam showed the most distributed crack pattern throughout loading. Numerous fine cracks developed progressively within the pure bending region, with no pronounced localization before the peak load. This distributed cracking enabled the tensile strain-hardening capacity of ECC to be mobilized over a comparatively long beam segment and resulted in the most stable deformation development among the tested configurations.

The comparison of the representative specimens therefore reveals a progressive change in the governing failure mechanism. For a mid-span connection without CFRP, failure is primarily controlled by damage at the mortise-and-tenon joint. CFRP strengthening suppresses this local joint damage but creates a new stiffness transition at the CFRP termination, where the critical crack may subsequently develop. Relocating the connection to the shear span further reduces its influence and allows the continuous pure-bending region to dominate the structural response. The resulting behavior becomes much closer to that of the monolithic ECC beam.

These observations show that connection behavior is controlled jointly by the epoxy-bonded interface, the keyed geometry, and the position of the connection relative to the bending-moment and shear-force distributions. Neither the experiments nor the adopted modeling strategy isolate an independent percentage contribution from adhesive bonding and geometric interlocking. The following sections therefore compare the response of the assembled connection system rather than assigning all resistance to the key geometry.

### 3.2. Structural Behavior of ECC Beams

Table 5 summarizes the peak load, corresponding mid-span deflection, and dominant failure mode of all 20 beams. Four unstrengthened mid-span-connected specimens exhibited joint opening, while ten CFRP-strengthened specimens developed localized ECC cracking at the CFRP termination. The remaining six specimens exhibited ECC flexural failure, including all three shear-span-connected beams and the monolithic reference specimen. These observations are considered together with the load–deflection curves when evaluating the effects of the principal test variables.

#### 3.2.1. Effect of ECC Strength

**Figure 10:**
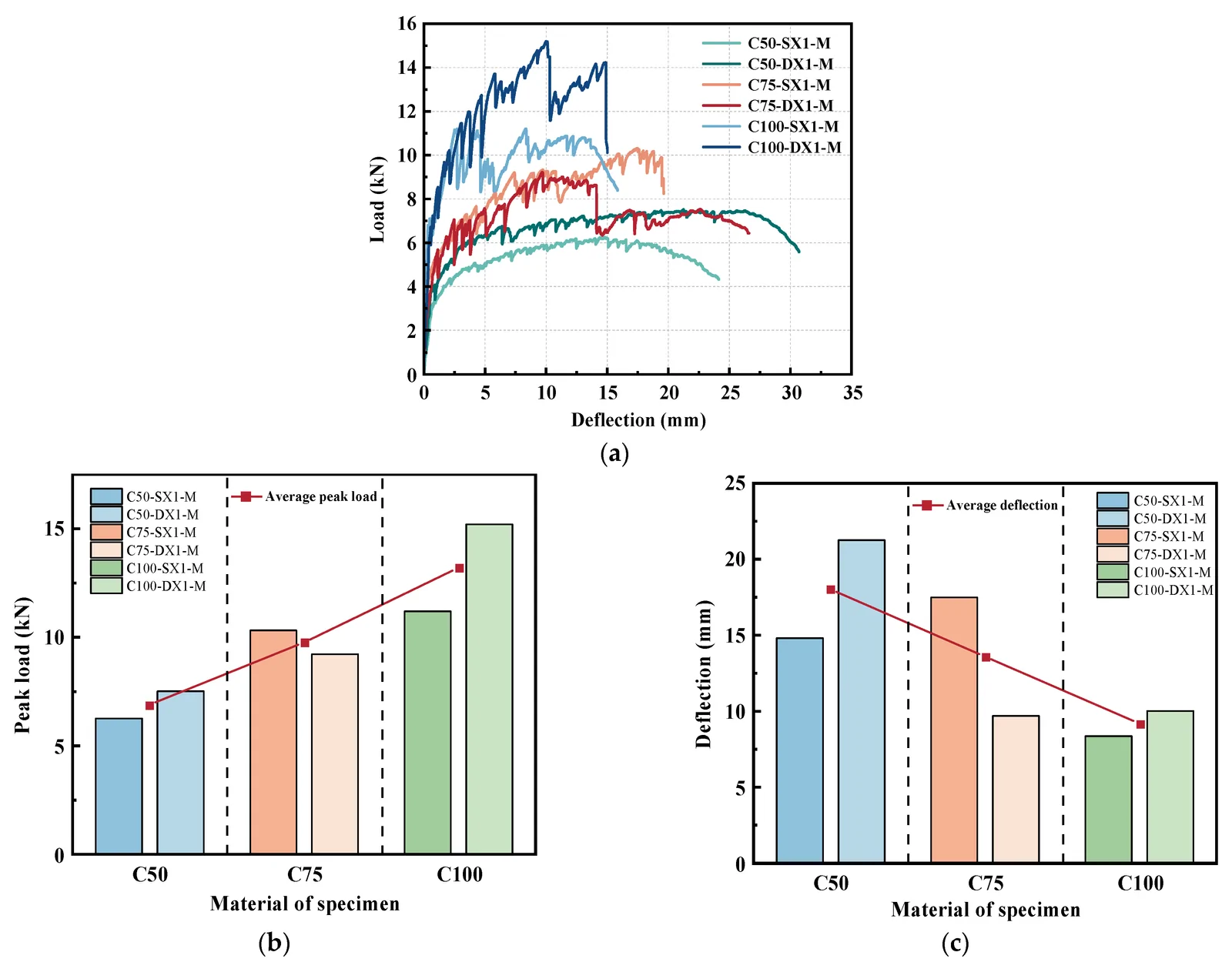
Effect of ECC strength grade on the flexural response: (**a**) load–deflection responses; (**b**) peak loads; (**c**) deflections at peak load.

Figure 10 compares six one-layer, X-oriented mid-span specimens manufactured with ECC50, ECC75, or ECC100. With geometry, strengthening, and joint location kept nominally identical within each straight- and dovetail-joint series, the differences characterize the response to changing ECC strength within these bonded connection configurations.

For the straight mortise-and-tenon beams, increasing the ECC grade from C50 to C75 increased the peak load by approximately 65%, whereas the further increase from C75 to C100 produced only an additional 9% improvement. The curve shape also changed considerably. The C50 and C75 specimens both exhibited relatively extended post-cracking hardening, and the deflection at peak load of C75 was approximately 18% larger than that of C50. In contrast, although C100 reached the highest load level, its corresponding deflection decreased by approximately 52% relative to C75, and the hardening stage became noticeably shorter. Therefore, for the straight connection, increasing the material strength from C50 to C75 improved both resistance and deformation development, whereas further increasing the strength mainly enhanced the load level at the expense of deformation capacity.

The dovetail beams exhibited a different development pattern. From C50 to C75, the peak load increased by only approximately 23%, while the deflection at peak load decreased by approximately 54%. When the strength was further increased to C100, however, the peak load increased by approximately 65%, reaching about twice that of the C50 specimen, whereas the deflection changed very little relative to C75. Thus, the major reduction in deformation occurred at the C50–C75 transition, while the major strength gain occurred at the C75–C100 transition. Compared with the straight joint, the effect of material strength on the dovetail connection was therefore more strongly reflected in the high-strength range.

These differences indicate that ECC strength does not affect the connected beams through a simple proportional increase in capacity. At the material level, the tensile strength increased progressively from ECC50 to ECC100, whereas the measured tensile strain capacity decreased from 7.86% to 5.11%. The higher tensile resistance increases the stress that can be sustained after cracking, while the reduced tensile strain capacity limits the deformation range available for continued multiple cracking. This explains the general transition from relatively long hardening responses at lower strength to higher but shorter responses at higher strength.

The different trends may also reflect differences in the load-transfer mechanisms of the two joint geometries. For straight joints, resistance depends primarily on the bonded interface and the remaining ECC section, so further increases in matrix strength may provide diminishing benefits once connection damage governs the response. In dovetail joints, inclined flanks redistribute transfer through local compression and shear. Higher matrix strength may increase resistance to bearing-induced crushing or chipping near the key roots, so a larger fraction of the material-strength gain can contribute to the global peak load. This interpretation is consistent with the larger peak-load increase observed in the ECC100 dovetail specimen, although local contact stresses were not measured directly.

#### 3.2.2. Effect of the Number of FRP Layers

**Figure 11:**
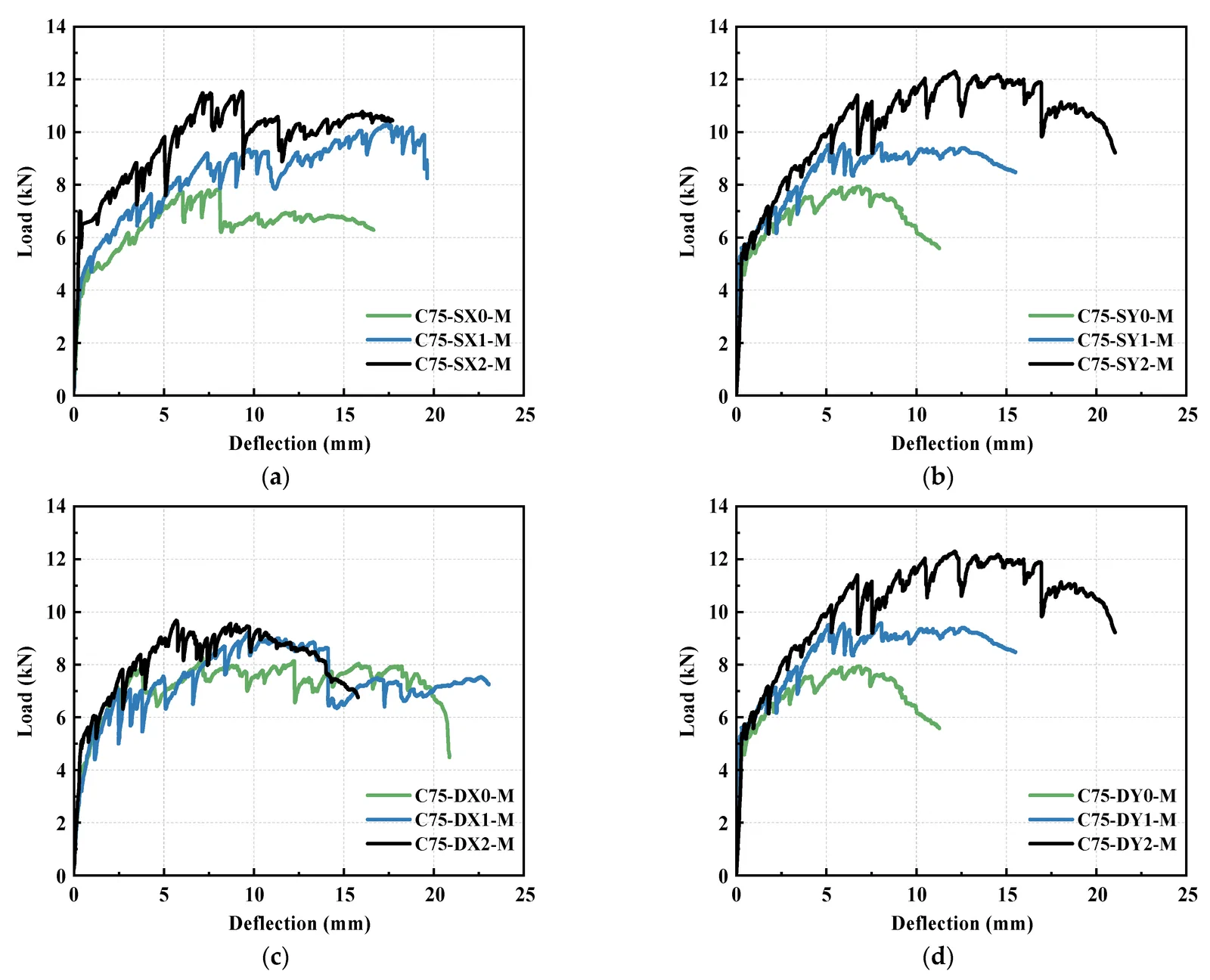
Effect of CFRP layer number on the load–deflection responses: (**a**) straight joint under X-direction loading; (**b**) straight joint under Y-direction loading; (**c**) dovetail joint under X-direction loading; (**d**) dovetail joint under Y-direction loading.

Figure 11 compares the load–deflection responses of the mid-span-connected beams with zero, one, and two CFRP layers. Overall, increasing the amount of CFRP produced a clear increase in peak load, whereas its effect on deformation was less uniform. For the straight connections, the strengthening effect was relatively consistent. Under X-direction loading, one and two CFRP layers increased the peak load by approximately 32% and 48%, respectively; under Y-direction loading, the corresponding increases were approximately 21% and 55%. The curves also show that the addition of CFRP generally extended the post-cracking response. In particular, the one-layer X-direction specimen exhibited a substantially longer hardening stage than the unstrengthened beam. However, increasing the CFRP from one to two layers did not always further increase deformation. For the X-direction straight joint, the deflection at peak load decreased markedly when the second layer was added, despite a further increase in peak load.

The dovetail specimens showed greater variability. Under X-direction loading, the capacity increases provided by one and two CFRP layers were only approximately 12% and 17%, respectively, considerably smaller than those of the straight specimens. Moreover, the two-layer specimen developed a shorter deformation range than the one-layer specimen. Under Y-direction loading, however, the second CFRP layer had a much stronger effect: the peak load increased by approximately 75% relative to the unstrengthened beam, accompanied by a substantial extension of the load–deflection curve. Thus, although CFRP strengthening was beneficial overall, its efficiency depended on the interaction between joint geometry and loading direction.

Considering all four mid-span configurations together, the average peak load increased by approximately 20% with one CFRP layer and by approximately 47% with two layers relative to the unstrengthened specimens. By comparison, the average deflection at peak load increased by approximately 49% with one layer but only 41% with two layers. The strength enhancement was therefore approximately monotonic, whereas the deformation improvement was not. This distinction is important because it shows that increasing CFRP amount does not simply produce a proportional improvement in the entire flexural response.

The observed failure modes help explain why increasing CFRP reinforcement did not consistently improve deformation capacity. Without CFRP, damage concentrated at the epoxy-bonded mid-span joint. CFRP wrapping restrained joint opening and enhanced load transfer across the connection, but the critical damage region shifted toward the CFRP termination as local strengthening increased. As summarized in Table 5, ten strengthened specimens exhibited localized ECC cracking at the CFRP termination, without obvious macroscopic CFRP debonding. Previous studies have identified CFRP-end separation and intermediate-crack debonding as important premature failure modes in externally strengthened concrete [28,29], while ECC–CFRP studies have shown how multiple cracking can influence interfacial behavior [35]. Unlike those interfacial failure modes, the present specimens developed their governing cracks in the ECC adjacent to the bonded CFRP sheet. The abrupt change in local flexural restraint may have promoted strain localization in this region, limiting further improvements in deformation capacity.

#### 3.2.3. Effect of Connection Type and Loading Direction

**Figure 12:**
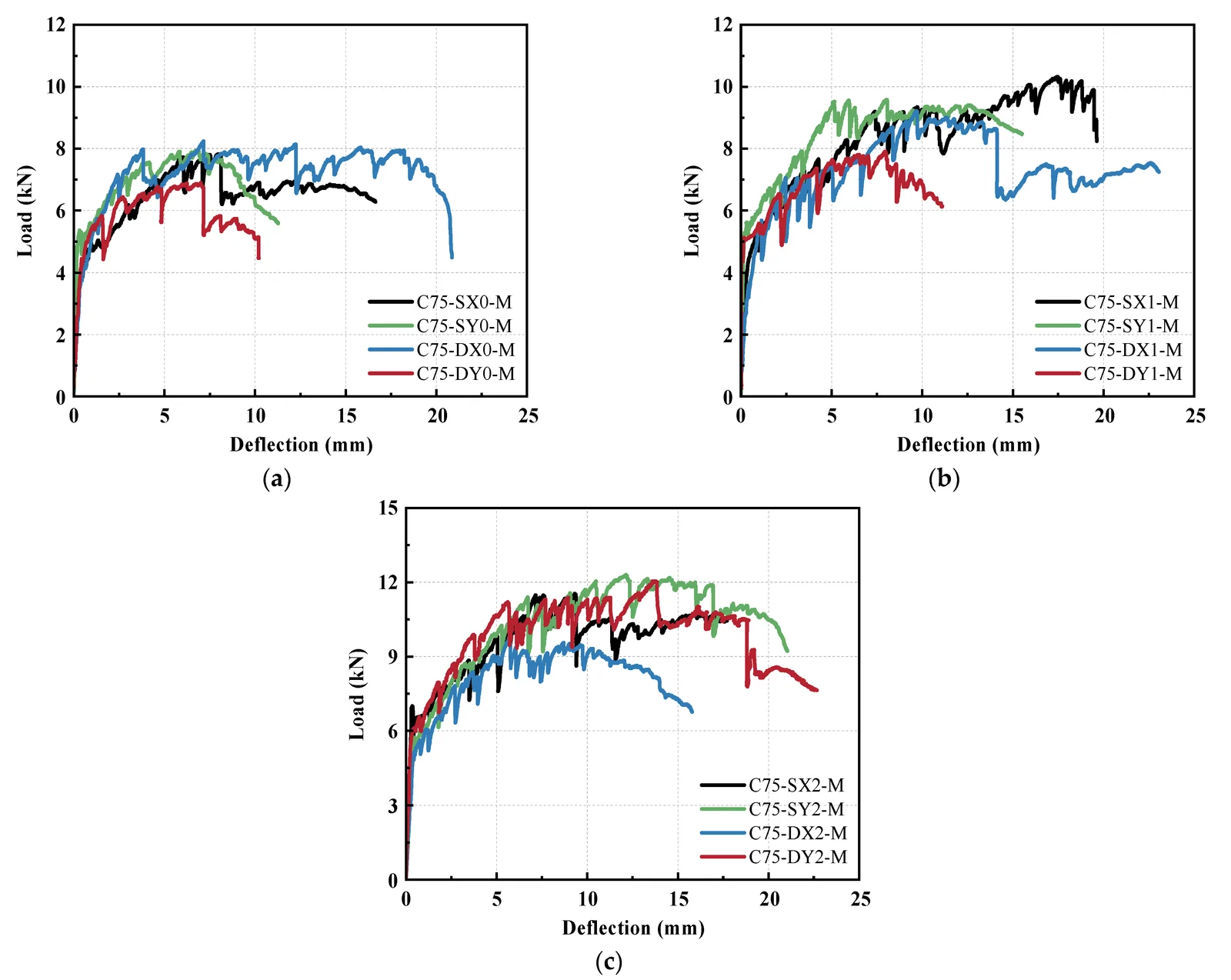
Effects of joint geometry and loading direction at different CFRP strengthening levels: (**a**) without CFRP; (**b**) one CFRP layer; (**c**) two CFRP layers.

Figure 12 reorganizes the experimental results to compare joint geometry and loading direction at the same CFRP strengthening level. Although the overall capacity difference between the two joint geometries was moderate, the straight joint exhibited a more stable response to loading direction. Across the zero-, one-, and two-layer CFRP groups, the mean peak loads of the straight-joint specimens were approximately 4.1%, 16.2%, and 9.8% higher, respectively, than those of the corresponding dovetail specimens. Since each combination was tested once, the smaller percentage differences should be treated as tendencies within this program rather than evidence of statistical superiority. The more complex dovetail shape did not provide a consistent capacity advantage over the straight shape.

Directional effects were more distinct. Peak-load differences between X- and Y-direction loading were approximately 1.5%, 7.4%, and 6.4% for straight joints, compared with 17.7%, 15.4%, and 21.7% for dovetail joints with zero, one, and two CFRP layers, respectively. The greater directional sensitivity of dovetail joints may be associated with their inclined contact surfaces, which alter the relative contributions of local bearing and adhesive bonding under different loading orientations. Previous direct-shear tests on precast segmental concrete joints have also demonstrated that both shear-key configuration and epoxy bonding influence joint resistance [24]. However, because all specimens in the present study were epoxy-bonded, the measured differences reflect the overall response of the bonded connection configurations rather than the independent contributions of geometric interlocking and adhesive bonding.

The influence of joint geometry and loading direction should nevertheless be interpreted together with the governing failure region. Without CFRP, the response was primarily controlled by damage at the bonded connection and the locally reduced section. After strengthening, the critical region progressively shifted toward the CFRP termination. Once either region governed the response, further differences associated with mortise-and-tenon geometry had a comparatively smaller influence on the global load–deflection behavior. Within the investigated configurations, the simpler straight joint therefore provided a more direction-insensitive load-transfer response without sacrificing flexural resistance.

#### 3.2.4. Effect of Connection Location

**Figure 13:**
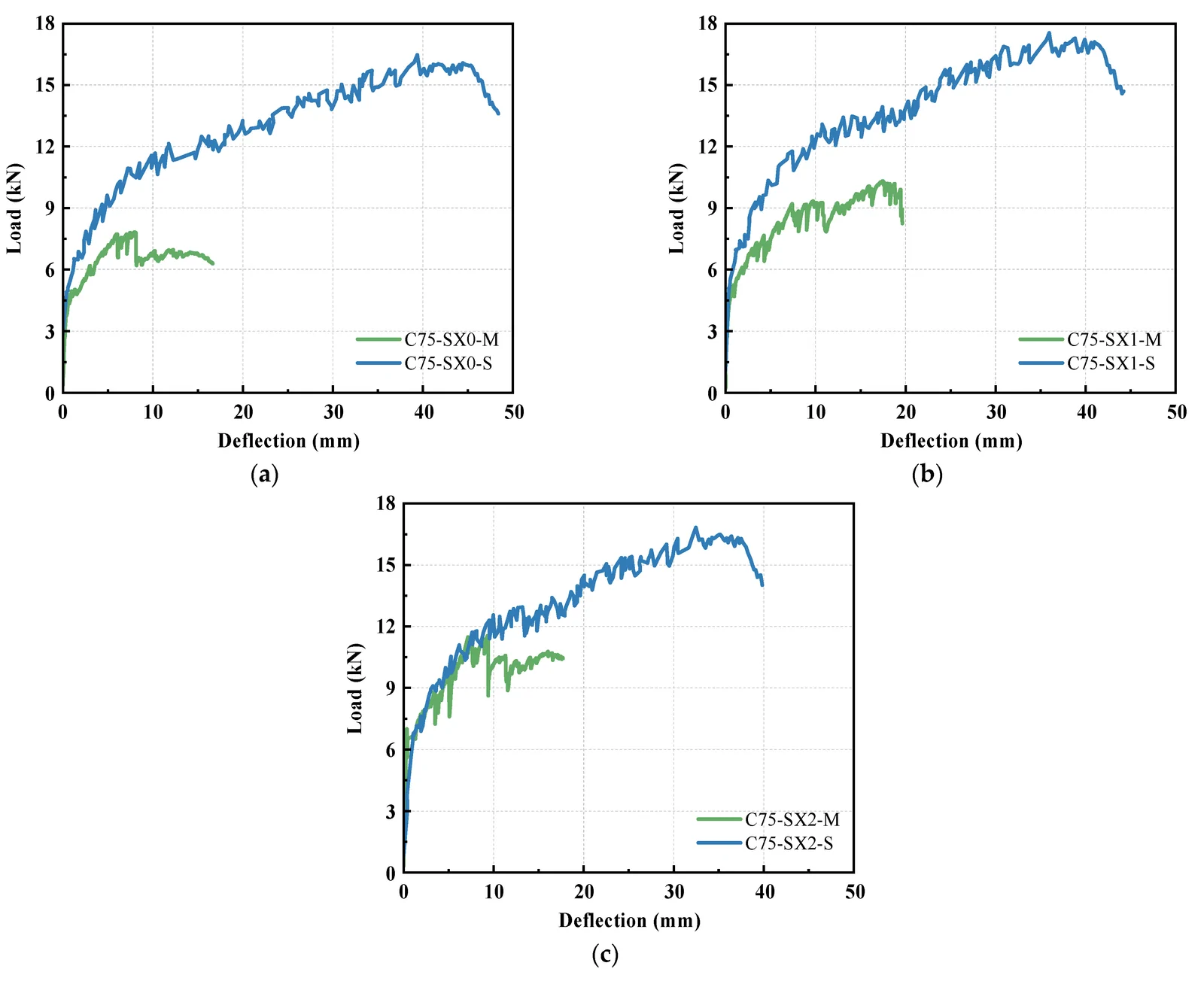
Effect of connection location on the load–deflection responses: (**a**) without CFRP; (**b**) one CFRP layer; (**c**) two CFRP layers.

Figure 13 compares the load–deflection responses of straight mortise-and-tenon beams with connections located at mid-span and within the shear span. Within the matched ECC75 straight-joint specimens under X-direction loading, relocating the connection produced large and consistent changes in structural response. For the unstrengthened beams, relocating the connection from mid-span to the shear span increased the peak load by approximately 111% and the corresponding deflection by 391%. With one CFRP layer, the increases were approximately 70% and 106%, respectively, while those with two layers were approximately 46% and 248%. These improvements were substantially greater than those achieved by increasing CFRP reinforcement alone within the corresponding mid-span configurations. It is worth noting that these percentages describe individual beam comparisons, not a statistical ranking of variables across a complete factorial design.

The load–deflection curves further reveal distinct deformation characteristics. The mid-span-connected beams exhibited relatively short post-cracking hardening stages, with deformation progressively localizing around the joint or CFRP termination. In contrast, the shear-span-connected beams sustained longer hardening stages and substantially greater deflections before reaching their peak loads. Their responses approached that of the monolithically cast reference beam, retaining approximately 89–94% of its peak load and 74–90% of its deflection at peak load. These results indicate that relocating the connection largely restored the global flexural response despite the presence of a discontinuous joint.

The improvement can be explained by the different internal-force demands at the connection and the resulting change in the governing failure region. Under symmetric four-point bending, the nominal bending moments at the mid-span and shear-span connections are 0.150P and 0.075P kN·m, respectively, where P is the total applied load in kN. The corresponding shear forces are zero and P/2 kN. Thus, relocating the connection reduces its bending-moment demand by 50% at the same load, although the shear-span joint is subjected to combined bending and shear. At mid-span, the geometric discontinuity and bonded interface coincide with the maximum-moment region, promoting premature damage localization and limiting the development of distributed cracking. Relocating the connection reduces this local flexural demand while preserving a continuous constant-moment region, allowing multiple cracking and fiber bridging to develop more extensively and thereby enhancing both load-bearing and deformation capacities. Notably, for the unstrengthened specimens, the peak load increased from 7.82 to 16.47 kN, while the nominal joint moment at peak load increased slightly from 1.173 to 1.235 kN·m. This distinction indicates that the higher global capacity is not simply associated with a lower joint moment at failure, but rather with delayed joint-controlled damage and fuller utilization of the continuous ECC flexural region. Despite the additional shear demand, the shear-span connection did not become the governing failure region in the investigated specimens.

The shear-span-connected beams also exhibited reduced sensitivity to CFRP strengthening. Increasing the CFRP from zero to one layer increased the peak load by only 6.5%, while two layers provided an increase of approximately 2.2% relative to the unstrengthened specimen. Meanwhile, the deflection at peak load decreased progressively with increasing CFRP layers. Unlike the mid-span connections, where CFRP substantially improved joint resistance, further strengthening of the shear-span connections offered limited additional capacity because the global response was primarily governed by the continuous ECC flexural region rather than premature connection failure.

## 4. Numerical Analysis

### 4.1. Finite-Element Modeling

**Figure 14:**
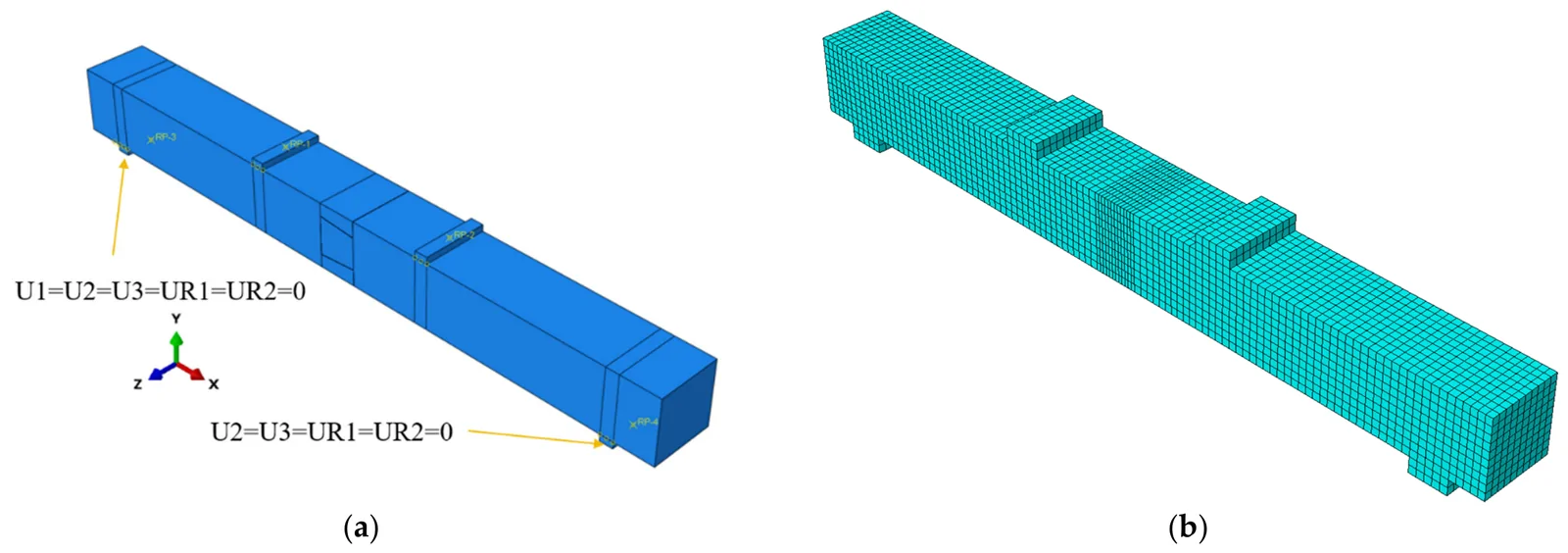
Numerical model: (**a**) boundary conditions; (**b**) mesh discretization.

Three-dimensional nonlinear finite-element models were established in ABAQUS/Standard using the Static, General procedure. The numerical geometry was consistent with the tested beams. The ECC matrix, loading and supporting blocks were modeled using eight-node linear brick elements with reduced integration (C3D8R), while the CFRP sheets were represented by four-node shell elements with reduced integration (S4R). The loading and supporting blocks were tied to the corresponding beam surfaces, and displacement-controlled loading was applied according to the experimental four-point bending configuration. As shown in Figure 14, a global mesh size of 10 mm was adopted, with local refinement to 5 mm around the mortise-and-tenon connection.

**Figure 15:**
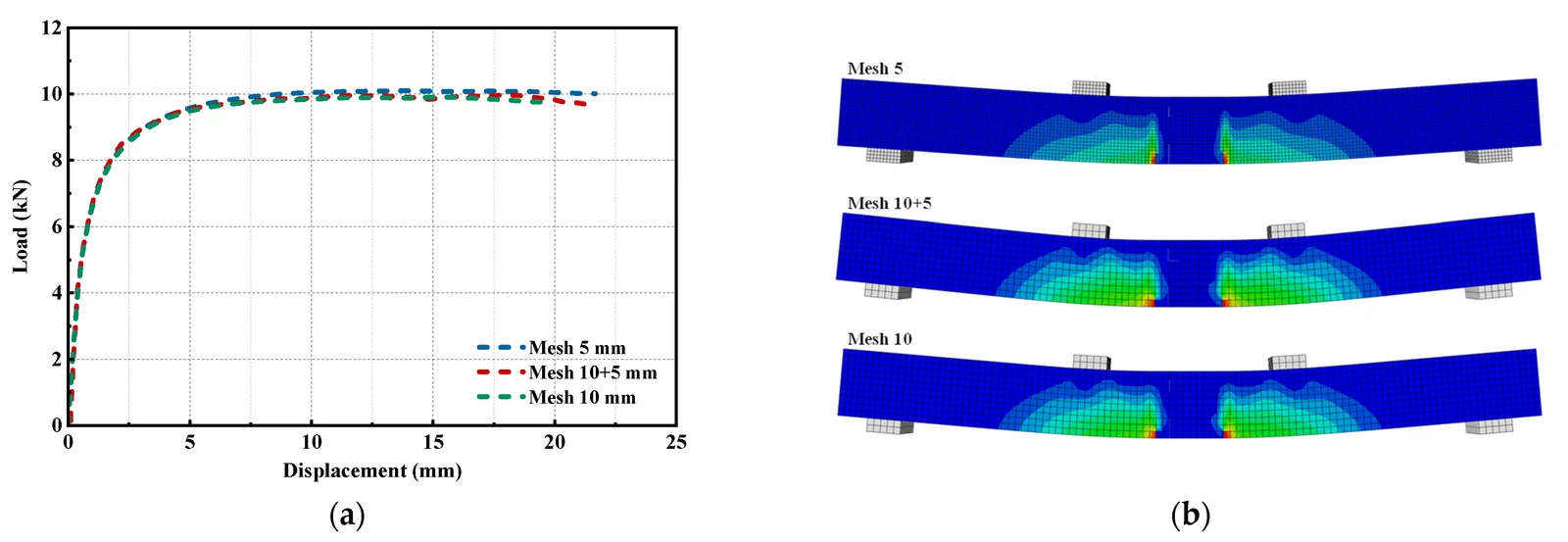
Mesh-sensitivity analysis: (**a**) load–deflection responses under different mesh configurations; (**b**) corresponding tensile damage distributions.

A mesh-sensitivity analysis was conducted to evaluate the influence of mesh refinement on the predicted structural response. Three mesh configurations were compared: a uniform 10 mm mesh, a 10 mm global mesh with local refinement to 5 mm around the connection, and a uniform 5 mm mesh. As shown in Figure 15a, the three load–deflection curves are nearly coincident. Figure 15b further shows that the three meshes predict similar tensile damage distributions, with damage consistently concentrated in the ECC adjacent to the connection. These results indicate that the predicted peak resistance and governing damage location are relatively insensitive to mesh refinement within the investigated range. Accordingly, the 10 mm global mesh with 5 mm local refinement was retained to balance computational efficiency and numerical accuracy.

The concrete damaged plasticity (CDP) formulation described ECC nonlinear response and stiffness degradation. For ECC75, the initial elastic modulus E_0_ and Poisson ratio were taken as 25 GPa and 0.20. The remaining CDP parameters were adopted with reference to previous numerical studies of ECC [36] and are summarized in Table 6. In uniaxial loading, CDP relates stress and inelastic deformation through σ_i_ = (1 − d_i_)E_0_(ε_i_ − ε_i_^pl^), where i = t or c and d_i_ denotes tensile or compressive stiffness damage (0 ≤ d_i_ < 1). The corresponding cracking or inelastic strain supplied to ABAQUS is $\tilde{\varepsilon_{i}}$ = ε_i_ − σ_i_/E_0_. For a specified damage curve, ABAQUS converts this quantity to plastic strain through ${\tilde{\varepsilon_{i}}}^{\mathrm{pl}}$ = $\tilde{\varepsilon_{i}}$ − [d_i_/(1 − d_i_)]σ_i_/E_0_. These relations distinguish the directly measured uniaxial response from the model-dependent damage variables, and require nonnegative, nondecreasing plastic strains [36].

**Figure 16:**
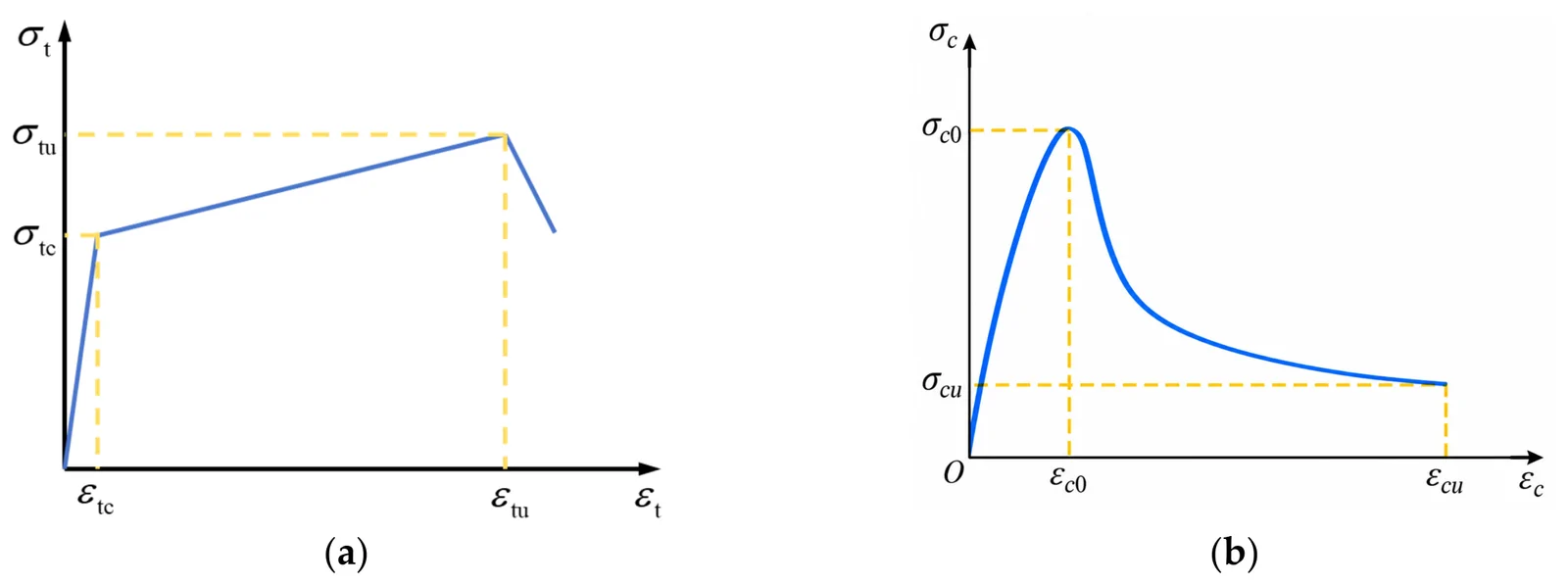
Constitutive models for ECC: (**a**) tensile; (**b**) compressive.

To reproduce the pronounced tensile strain-hardening behavior of ECC, the tensile constitutive relationship was represented by the trilinear model proposed by Han et al. [37]. For ECC75, the first-cracking stress and corresponding strain were 4.9 MPa and 0.026%, respectively, while the tensile strength and corresponding tensile strain were 8.4 MPa and 6.71%. The compressive relationship followed the model proposed by Xu et al. [38]. The peak stress was 74.34 MPa at a strain of approximately 0.34%. The residual stress was set to 20% of the peak stress (14.87 MPa) at a strain of approximately 1.0%. The characteristic constitutive parameters are summarized in Table 7, and the corresponding tensile and compressive relationships are illustrated in Figure 16.

The CFRP sheets were modeled using S4R shell elements with an elastic modulus of 230 GPa, a Poisson ratio of 0.20, and a thickness of 0.167 mm per layer. A linear-elastic constitutive model was adopted, and the CFRP–ECC interface was defined using a tie constraint because no obvious separation within the bonded region was observed during the tests.

The epoxy-bonded ECC–ECC interface was modeled by a surface-based cohesive–friction formulation, using the approach adopted for grouted mortise-and-tenon concrete interfaces by Yang et al. [39]. The cohesive parameters represent the effective bonded ECC–adhesive–ECC assembly, not the bulk tensile or shear behavior of neat Sikadur-330CN. Before damage, normal and tangential traction were related linearly to the relative surface separations. As shown in Table 8, normal and tangential stiffnesses of 1.0 × 10^4^ and 10 MPa/mm, respectively, were adopted to represent high pre-damage normal restraint and finite interface slip compliance. Damage initiated under the quadratic nominal-stress criterion shown in Equation (1). To determine the tensile bond strength of the epoxy-bonded ECC interface, splitting tensile tests were conducted on four 40 × 40 × 40 mm^3^ cubic specimens, each consisting of two cast ECC75 segments bonded with Sikadur-330 CN epoxy adhesive. The testing approach was based on the splitting tensile method for epoxy-bonded concrete interfaces reported by Newlands et al. [40], using a cubic specimen configuration similar to that adopted by Wolfs et al. [41]. The specimens were tested after approximately one month of curing, and the measured mean interfacial splitting tensile strength was 2.98 MPa. Accordingly, a nominal normal cohesive strength of 3 MPa was adopted in the numerical model. For simplicity, the nominal strengths in the two tangential directions were also set to 3 MPa, with displacement-based linear damage evolution to an effective separation of 1 mm. After cohesive degradation, hard normal contact prevented interpenetration and a friction coefficient of 0.6 represented residual sliding resistance. Parameters other than the nominal interface strength were selected from a precedent cohesive–friction modeling formulation [39] and should be regarded as effective model inputs, rather than directly measured properties of the adhesive layer. The validation in Section 4.2 supports their combined structural-level adequacy for the studied specimens, but does not identify each interface parameter uniquely.

(1) $${\left(\frac{\langle t_{n}\rangle }{t_{n}^{0}}\right)}^{2}+{\left(\frac{t_{s}}{t_{s}^{0}}\right)}^{2}+{\left(\frac{t_{t}}{t_{t}^{0}}\right)}^{2}=1$$

where *t*_n_, *t*_s_, and *t*_t_ are the nominal tractions in the normal and two tangential directions, respectively. The corresponding peak nominal stresses $t_{n}^{0}$, $t_{s}^{0}$, and $t_{t}^{0}$ are the corresponding interfacial strengths. The Macaulay bracket ensures that compressive normal stress does not initiate cohesive damage. After damage initiation, a linear displacement-based softening law was adopted until complete loss of cohesive traction. Hard contact was defined in the normal direction to prevent penetration, while tangential sliding was governed by penalty friction. This cohesive–friction formulation therefore allows the joint load-transfer mechanism to evolve from adhesive bonding to contact, geometric interlocking, and friction as interfacial damage develops.

### 4.2. Validation of the Numerical Model

**Figure 17:**
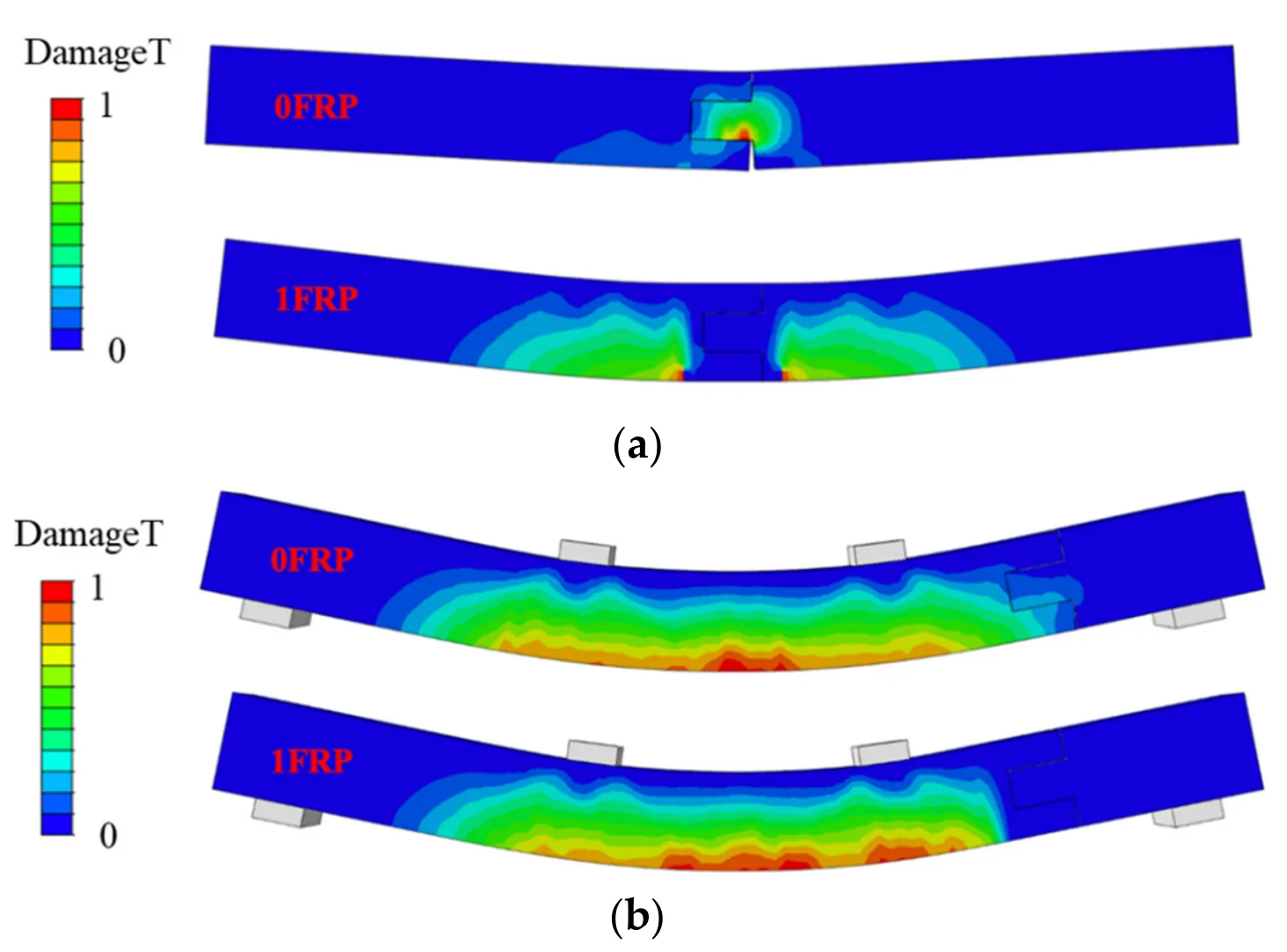
Tensile damage contours of representative connected beams: (**a**) mid-span-connected beams; (**b**) shear-span-connected beams.

The numerical model was validated in terms of both damage distribution and global load–deflection response. Figure 17 presents the tensile damage contours (DAMAGET) of representative mid-span- and shear-span-connected beams. For the unstrengthened mid-span connection, tensile damage was strongly localized around the mortise-and-tenon region, indicating that the geometric discontinuity and adhesive interface governed the local response. After CFRP strengthening, damage within the wrapped joint was markedly suppressed, while pronounced damage developed near the CFRP termination. This shift in the critical region agrees with the experimental observation that CFRP restrained opening at the original joint but promoted crack localization adjacent to the strengthened zone.

A different damage pattern was obtained for the shear-span-connected beams. The connection region remained comparatively less damaged, while tensile damage developed predominantly in the continuous flexural region. Moving the connection away from the maximum-moment region therefore reduced its influence on the global flexural response and allowed the strain-hardening behavior of the continuous ECC segment to be more fully mobilized. Overall, the numerical damage patterns reproduced the experimentally observed changes in damage localization associated with connection location and CFRP strengthening.

**Figure 18:**
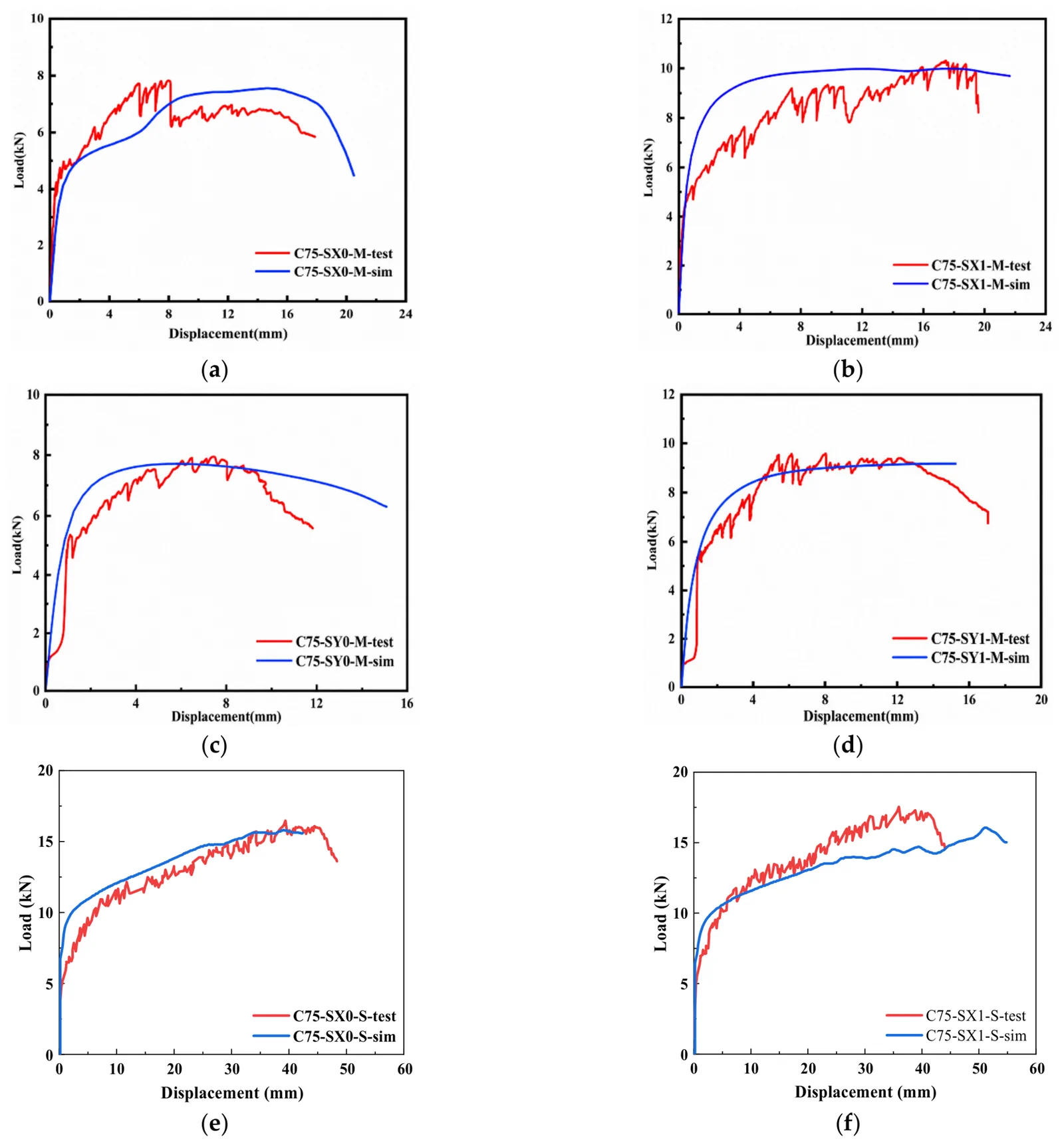
Comparison between experimental and numerical load–deflection responses of representative specimens: (**a**) C75-SX0-M; (**b**) C75-SX1-M; (**c**) C75-SY0-M; (**d**) C75-SY1-M; (**e**) C75-SX0-S; (**f**) C75-SX1-S.

Figure 18 compares six numerical and experimental load–deflection responses. The numerical peak loads differed from the experimental values by −3.4% to −8.6%, with a mean absolute percentage error of 4.55% and an RMSE of 0.73 kN (Table 9). The mean absolute percentage error in peak deflection was higher, at 34.44%, indicating greater uncertainty in the prediction of deformation at the maximum load. This is particularly apparent where the numerical curve has a broad load plateau: the location of a single numerical maximum can shift markedly along that plateau even though the overall load level remains near the experimental one. The initial rising branches and changes in governing damage location are reproduced qualitatively, although the simplified material and interface models cannot fully capture the localized cracking and deformation observed experimentally. In particular, the tie constraint adopted for the CFRP–ECC interface excludes local bond-slip, interfacial cracking, and peeling near the sheet termination. These mechanisms may redistribute interfacial stresses and reduce effective anchorage, so the assumption of perfect bonding may overestimate CFRP restraint and affect the predicted load–deflection response, particularly the peak deflection and post-peak behavior [28,29,35].

### 4.3. Parametric Analysis

A numerical sensitivity study was conducted for ECC75 straight mid-span joints to evaluate local CFRP strengthening, holding all other modeled parameters constant. The numerical comparisons are conditioned on the adopted ECC constitutive law, cohesive-contact properties, and fully bonded CFRP–ECC interface and they do not constitute a calibrated design optimization across ECC grades or joint geometries.

**Figure 19:**
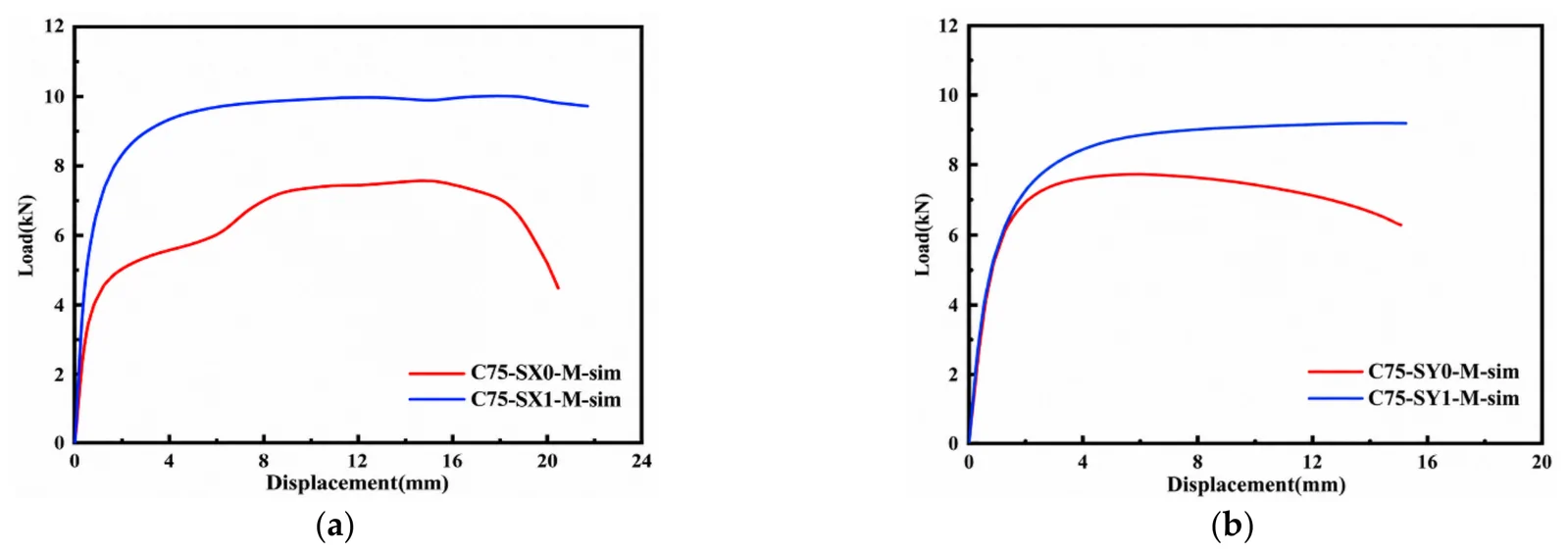

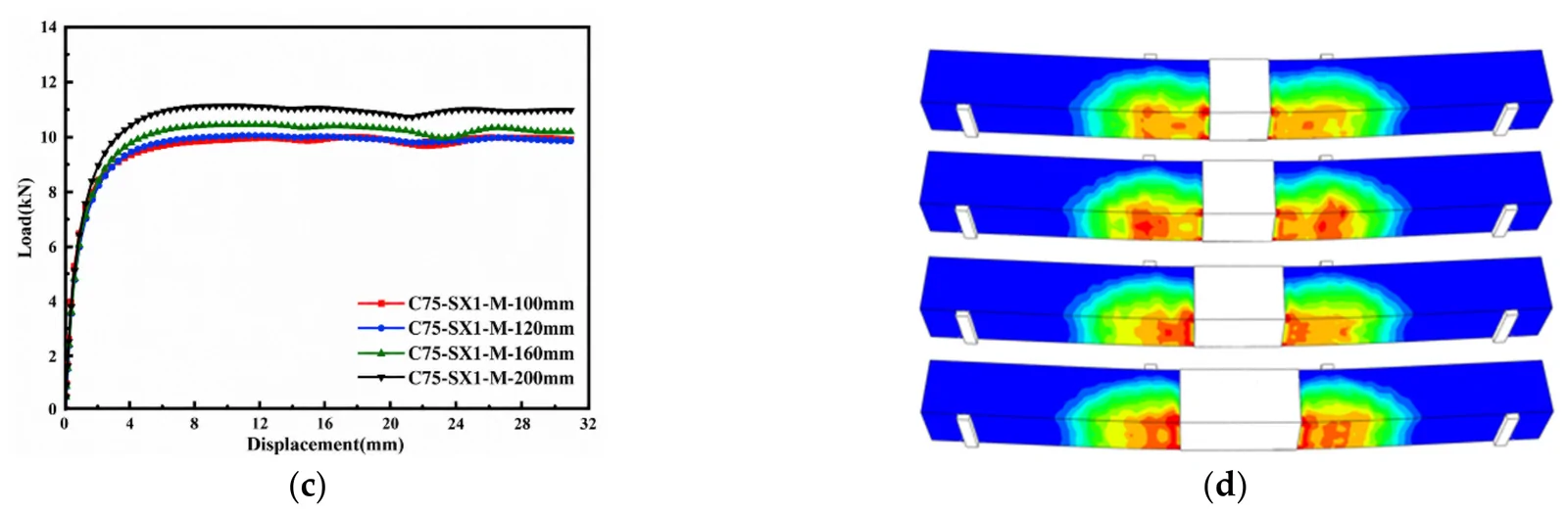
Numerical parametric analysis of CFRP strengthening: (**a**) effect of CFRP strengthening under X-direction loading; (**b**) effect of CFRP strengthening under Y-direction loading; (**c**) load–deflection responses with different CFRP widths; (**d**) tensile damage contours with different CFRP widths.

Figure 19a,b compare the numerical responses of the unstrengthened and one-layer CFRP-strengthened beams under the two loading directions. Without CFRP, the predicted peak loads were 7.21 and 7.43 kN, respectively. After one CFRP layer was applied, the corresponding values increased to 9.83 and 9.11 kN, representing increases of 36.3% and 22.6%, respectively, or approximately 29% on average. These strengthening trends agree with the experimental direction, although the magnitude differs because of model simplifications, including homogenized ECC cracking and perfect CFRP adhesion. Such agreement supports a mechanistic interpretation of enhanced restraint, rather than a claim that the individual interface parameters have been independently identified.

The influence of CFRP wrapping width was further investigated using widths of 100, 120, 160, and 200 mm, as shown in Figure 19c,d. The corresponding peak loads were approximately 9.9, 10.1, 10.5, and 11.1 kN, respectively. Increasing the CFRP width from 100 to 200 mm therefore increased the peak load by only about 12%, despite doubling the strengthened width. In addition, the initial portions of the four load–deflection curves were nearly coincident, indicating that the CFRP width had little influence on the initial flexural stiffness. The principal effect of increasing the wrapping width was thus a moderate increase in the post-cracking load-carrying level rather than a fundamental change in the global response.

The stronger influence of CFRP width on damage location than on peak load can be understood through the redistribution of tensile transfer. As the modeled width increased, the strengthened region restrained deformation around the epoxy-bonded joint, while the adjacent unstrengthened ECC became the dominant deformation zone (Figure 19d). The predicted 12% peak-load gain for doubling width from 100 to 200 mm therefore represents diminishing incremental resistance in this specific numerical configuration. Since the tie constraint excludes peeling and bond-slip, extending the bonded length may be more effective in the simulation than in practice, and the magnitude of the benefit or the precise failure location may change when interface debonding is permitted. These results motivate experimental evaluation of CFRP anchorage length and termination detailing.

## 5. Conclusions

This study investigated the flexural behavior of rebar-free ECC beams assembled using mortise-and-tenon connections through 20 four-point bending tests and nonlinear finite-element analysis. The effects of ECC strength, CFRP strengthening, connection geometry, loading direction, and connection location were evaluated. The main conclusions are as follows:

(1) The mortise-and-tenon connection significantly affected the development and localization of flexural damage when located in the maximum-moment region. In unstrengthened mid-span-connected beams, cracking and interface damage progressively concentrated around the joint, limiting the development of distributed ECC cracking. CFRP restrained opening of the original connection and delayed joint-controlled failure, but the governing crack subsequently shifted toward the CFRP termination because of the local stiffness transition.
(2) CFRP strengthening consistently improved the load-bearing capacity of mid-span-connected beams. Compared with the unstrengthened specimens, one and two CFRP layers increased the average peak load by approximately 20% and 47%, respectively. The corresponding improvement in deformation was less systematic: one CFRP layer generally extended the post-cracking hardening stage, whereas further strengthening could promote earlier localization adjacent to the strengthened region. Therefore, increasing CFRP amount does not necessarily produce a proportional improvement in the overall flexural response.
(3) Increasing ECC strength generally enhanced flexural resistance but reduced deformation capacity, with the magnitude of this trade-off depending on joint geometry. At the same CFRP strengthening level, straight joints maintained comparable or moderately higher peak loads than dovetail joints and exhibited substantially lower sensitivity to loading direction. The X-Y peak-load differences were only 1.5–7.4% for straight joints, compared with 15.4–21.7% for dovetail joints. Thus, the simpler straight geometry provided a more direction-stable load-transfer response without a systematic loss of flexural resistance.
(4) Connection location had a substantial influence on the response within the investigated beams. Moving the joint from the maximum-moment region to the shear span substantially extended the post-cracking hardening stage and restored a response close to that of the monolithic beam. The shear-span-connected specimens retained approximately 89–94% of the reference peak load and 74–90% of its deflection at peak load. Moreover, once the connection was located in the shear span, additional CFRP produced only limited changes in peak load, indicating that reducing the demand on the joint is more effective than relying solely on increased local strengthening.
(5) The numerical models captured the broad load–deflection shape, peak resistance, and experimentally observed shift in damage region. The conditional numerical comparison predicted an approximately 12% peak-load gain when CFRP width increased from 100 to 200 mm, while moving damage toward the CFRP termination. Given the idealized CFRP tie constraint, this result should be treated as a sensitivity trend, not a validated detailing requirement.

The present evidence is limited to 100 × 100 mm^2^ beams tested monotonically, one structural specimen per configuration, two keyed geometries, and epoxy-bonded interfaces. The program lacks corresponding ECC50 and ECC100 monolithic controls, unbonded joint controls, and repeated structural tests. The FE results are further affected by the assumed bond and mesh-dependent localization. Before engineering generalization, further work should examine replicated and larger-scale tests, dry-versus-bonded controls, measured interface traction–separation properties, cyclic/fatigue loading, environmental aging, and the sensitivity of CFRP termination performance to bond-slip and peeling.

## Figures and Tables

**Table 1:** Mix proportion of ECC (kg/m^3^).

Number	Cement	GGBS	SF	FA	Sand	WRA	Water	PE
ECC50	436	419	109	109	428	0.5	429	16
ECC75	514	494	129	129	504	2	341	16
ECC100	600	575	150	150	590	8.5	236	18

**Table 2:** Measured ECC mechanical properties (mean ± sample standard deviation; *n* = 3).

Material	Compressive Strength (MPa)	Tensile Strength (MPa)	Tensile Strain (%)
ECC50	50.72 ± 2.08	6.15 ± 1.38	7.86 ± 1.10
ECC75	74.34 ± 1.40	8.41 ± 1.42	6.71 ± 1.12
ECC100	96.76 ± 5.07	11.38 ± 1.40	5.11 ± 1.02

**Table 3:** Design specifications of all beams.

Number	ECC Strength (MPa)	Connection Form	Loading Direction	FRP Layer	Connection Location
C75-REF	75	-	-	-	-
C75-SX0-M	75	Straight	X	0	Mid
C75-SX1-M	75	Straight	X	1	Mid
C75-SX2-M	75	Straight	X	2	Mid
C75-DX0-M	75	Dovetail	X	0	Mid
C75-DX1-M	75	Dovetail	X	1	Mid
C75-DX2-M	75	Dovetail	X	2	Mid
C75-SX0-S	75	Straight	X	0	Shear
C75-SX1-S	75	Straight	X	1	Shear
C75-SX2-S	75	Straight	X	2	Shear
C75-SY0-M	75	Straight	Y	0	Mid
C75-SY1-M	75	Straight	Y	1	Mid
C75-SY2-M	75	Straight	Y	2	Mid
C75-DY0-M	75	Dovetail	Y	0	Mid
C75-DY1-M	75	Dovetail	Y	1	Mid
C75-DY2-M	75	Dovetail	Y	2	Mid
C50-SX1-M	50	Straight	X	1	Mid
C100-SX1-M	100	Straight	X	1	Mid
C50-DX1-M	50	Dovetail	X	1	Mid
C100-DX1-M	100	Dovetail	X	1	Mid

**Table 4:** Loads and mid-span deflections at five characteristic loading stages of representative specimens.

Specimen	Stage I (40%)	Stage II (60%)	Stage III (80%)	Stage IV (Peak)	Stage V (Post-Peak)
C75-SX0-M	3.13/0.27	4.69/0.66	6.26/3.59	7.82/8.02	6.28/16.65
C75-SX1-M	4.13/0.36	6.19/2.15	8.26/5.77	10.32/17.49	8.24/19.63
C75-SX1-S	7.02/1.42	10.52/5.77	14.03/20.15	17.54/35.95	14.69/44.23
C75-REF	7.43/1.24	11.15/6.64	14.86/28.27	18.58/43.99	15.38/51.06

Note: Each cell reports the applied load, P (kN), and the corresponding mid-span deflection, δ (mm), in the format P/δ.

**Table 5:** Peak responses and observed failure modes of beams.

Number	*P_u_* (kN)	∆*_u_* (mm)	∆*_u_*/*L*	Failure Mode
C75-SX0-M	7.82	8.02	1/112	Joint opening
C75-SX1-M	10.32	17.49	1/51	ECC cracking at CFRP termination
C75-SX2-M	11.54	9.32	1/97	ECC cracking at CFRP termination
C75-DX0-M	8.24	7.16	1/126	Joint opening
C75-DX1-M	9.22	9.69	1/93	ECC cracking at CFRP termination
C75-DX2-M	9.68	5.68	1/158	ECC flexural failure
C75-SY0-M	7.94	6.86	1/131	Joint opening
C75-SY1-M	9.58	8.05	1/112	ECC cracking at CFRP termination
C75-SY2-M	12.30	12.14	1/74	ECC cracking at CFRP termination
C75-DY0-M	6.90	7.00	1/129	Joint opening
C75-DY1-M	7.90	7.98	1/113	ECC flexural failure
C75-DY2-M	12.04	13.78	1/65	ECC cracking at CFRP termination
C50-SX1-M	6.26	14.80	1/61	ECC cracking at CFRP termination
C50-DX1-M	7.52	21.26	1/42	ECC cracking at CFRP termination
C100-SX1-M	11.20	8.37	1/108	ECC cracking at CFRP termination
C100-DX1-M	15.20	10.01	1/90	ECC cracking at CFRP termination
C75-SX0-S	16.47	39.37	1/23	ECC flexural failure
C75-SX1-S	17.54	35.95	1/25	ECC flexural failure
C75-SX2-S	16.83	32.45	1/28	ECC flexural failure
C75-REF	18.58	43.99	1/20	ECC flexural failure

*P_u_*: peak load; ∆*_u_*: deflection at peak load.

**Table 6:** Typical parameters of ECC.

Poisson’s Ratio	Dilation Angle (◦)	Eccentricity	*σ_b_*_0_/*σ_c_*_0_	*K_c_*	Viscosity Parameter
0.2	30	0.1	1.16	0.667	0.0005

**Table 7:** The characteristic points of the constitutive model of the ECC.

	Elastic Modulus *E*_0_ (MPa)	Cracking Tensile Stress *σ_tc_* (MPa)	Cracking Tensile Strain *ε_tc_* (%)	Peak Tensile Strength *σ_tu_* (MPa)	Strain at Peak Tensile Stress *ε_tu_* (%)	Peak Compressive Strength *σ_c_*_0_ (MPa)	Strain at Peak Compressive Stress *ε_c_*_0_ (%)	Residual Compressive Stress *σ_cu_* (MPa)	Strain at Residual Compressive Stress *ε_cu_* (%)
ECC75	25,000	4.90	0.026	8.41	6.71	74.34	0.34	14.87	1.00

**Table 8:** Numerical parameters of the mortise-and-tenon interface.

*K*_nn_ (MPa/mm)	*K*_ss_ = *K*_tt_ (MPa/mm)	*t*_n_^0^ = *t*_s_^0^ = *t*_t_^0^ (MPa)	Damage Evolution	*δ*_f_ (mm)	*μ*
1.0 × 10^4^	10	3	Linear, displacement-based	1	0.6

**Table 9:** Experimental and numerical peak-response comparison.

Beam	P_exp_ (kN)	P_FE_ (kN)	(P_FE_ − P_exp_)/P_exp_ (%)	δ_exp_ (mm)	δ_FE_ (mm)	(δ_FE_ − δ_exp_)/δ_exp_ (%)
C75-SX0-M	7.82	7.53	−3.7	8.02	14.43	+79.9
C75-SX1-M	10.32	9.97	−3.4	17.49	18.08	+3.4
C75-SY0-M	7.94	7.67	−3.4	6.86	5.09	−25.8
C75-SY1-M	9.58	9.19	−4.1	8.05	12.51	+55.4
C75-SX0-S	16.47	15.79	−4.1	39.37	38.92	−1.1
C75-SX1-S	17.54	16.03	−8.6	35.95	50.68	+41.0

## Data Availability

The original contributions presented in this study are included in the article. Further inquiries can be directed to the corresponding author.
